# Implementation strategy for an antibiotic stewardship bundle to promote optimal treatment choices in neonates with suspected early-onset sepsis (Protect-Neo): a study protocol for a multicentre, prospective interrupted time series and before-after study

**DOI:** 10.1136/bmjopen-2025-103368

**Published:** 2025-11-04

**Authors:** Liesanne E J van Veen, Gerdien A Tramper-Stranders, Niek B Achten, Frans B Plötz, Annemarie M C van Rossum, Erwin Ista

**Affiliations:** 1 Department of Paediatrics, Franciscus Gasthuis, Rotterdam, The Netherlands; 2 Department of Paediatrics, Sophia Children Hospital, Erasmus MC University Medical Center Rotterdam, Rotterdam, The Netherlands; 3 Department of Paediatrics, Tergooi Medical Centre, Hilversum, The Netherlands; 4 Department of Paediatrics, Emma Children’s Hospital, Amsterdam UMC Locatie AMC, Amsterdam, The Netherlands; 5 Department of Internal Medicine, Erasmus MC University Medical Center Rotterdam, Rotterdam, The Netherlands; 6 Department of Neonatal and Paediatric Intensive Care, Sophia Children Hospital, Erasmus MC University Medical Center Rotterdam, Rotterdam, The Netherlands

**Keywords:** INFECTIOUS DISEASES, Antibiotics, NEONATOLOGY, Sepsis

## Abstract

**Abstract:**

**Background:**

Several antibiotic stewardship interventions have been proven effective and safe for reducing the high number of antibiotic prescriptions in late preterm and term neonates at risk of early-onset sepsis (EOS). For successful translation of EOS interventions to clinical practice, implementation strategies should be employed targeting stakeholders. The primary aim of this study is to assess the impact of implementing an antibiotic stewardship bundle, including the EOS calculator, procalcitonin-guided therapy and intravenous-to-oral switch therapy on antibiotic exposure for EOS in Dutch secondary hospitals. Secondary aims are to examine additional clinical outcomes and implementation outcomes.

**Methods and analysis:**

We will conduct a multicentre, prospective implementation study with interrupted time series and before-after analyses at the paediatric or specialised neonatal departments of 11 Dutch secondary hospitals and their surrounding neonatal care networks. A multimodal implementation strategy, designed using Implementation Mapping, is employed to facilitate implementation. The study population is twofold: (1) neonates born at 34 weeks of gestation or later with suspected EOS that will receive intervention-related care and (2) paediatricians, paediatric residents, neonatal nurses, maternity nurses and parents who are the focus of the implementation strategies. The primary outcome is days of antibiotic therapy per 1000 live-born neonates, which will be evaluated using interrupted time series analysis as well as before-after comparison. Secondary clinical outcomes will be assessed by comparing clinical data from the 12 months pre-implementation and post implementation. Implementation outcomes are adoption, fidelity, feasibility and acceptability of the interventions and fidelity and appropriateness of the implementation strategies. Implementation outcomes will be assessed using both qualitative and quantitative methods, including surveys, individual interviews and focus group interviews. A mixed-methods approach will be used to integrate clinical and implementation outcomes.

**Ethics and dissemination:**

The Medical Ethics Committee United (MEC-U) declared (reference: W24.132) that this study does not fall under the Dutch Medical Research Involving Human Subjects Act (WMO). Subsequently, ethical approval was granted by the Scientific Committee of the Franciscus Hospital (T110). The scientific committees of all participating sites adopted this decision and granted permission for local conduct of the study. As electronic health record data are sampled retrospectively and anonymously, a waiver of consent was given to collect these data. Informed consent will be obtained from participants completing surveys or taking part in interviews and focus group discussions. The findings will be disseminated through journal publications and conference presentations. Furthermore, practice and policy recommendations will be collaboratively developed with partner organisations.

**Trial registration number:**

NCT06845332.

**STRENGTHS AND LIMITATIONS OF THIS STUDY**

- This study utilises implementation strategies that were systematically developed via Implementation Mapping, based on comprehensive stakeholder input.
- The study’s practical, real-world approach reflects daily clinical practice, enhancing applicability outside controlled research settings.
- Implementing three interventions simultaneously allows insight into their interaction and combined effects.
- Automated extraction of data through Business Intelligence units limits the capture of detailed patient-level data.
- Lack of randomisation restricts causal conclusions, but the mixed-methods approach provides valuable insights into the impact and appropriateness of the strategies.

## Introduction

 Empirical treatment of neonates with suspected early-onset sepsis (EOS) largely contributes to the widespread use of intravenous antibiotics and hospitalisation in the first days of life.^1^,^3^ Given the increasing knowledge on adverse short- and long-term effects of early antibiotic use and the rising relevance of delivering appropriate care, there is a clear need to implement evidence-based strategies that focus on considerate use of antibiotic therapy and stimulate patient-centred care.^4^,^8^ This is especially evident given that current antibiotic prescription rates are disproportionate to the actual incidence of culture-proven EOS, highlighting the opportunity to optimise antimicrobial use.^1 9^

Recently, several antibiotic stewardship interventions have been studied to answer this need, intervening at different stages of managing neonates at risk for EOS.^10^,^14^ Three interventions with high-quality evidence for late preterm and term neonates are the EOS calculator, procalcitonin (PCT)-guided therapy and intravenous (IV)-to-oral switch therapy. First, utilisation of the EOS calculator, developed by Kaiser Permanente, has shown to reduce up to 44% of antibiotic prescriptions in neonates at increased risk of EOS compared with the conventional risk categorisation approach.^10 15 16^ Second, a PCT-guided treatment regimen showed the potential to significantly reduce duration of empiric antibiotic treatment in neonates at low or medium risk of infection.^12^ Third, in neonates in which bacterial infection cannot be ruled out, despite a negative blood culture, switching from IV to oral antibiotics after 2 days (IV-to-oral switch therapy) was found to be as safe and effective as a 7-day intravenous treatment regimen.^11 17^ These interventions promote considerate use of antibiotics and patient-friendly care, ultimately improving the quality of life of neonates and their families. Additionally, reducing hospitalisation may contribute to cost savings in healthcare and increase the availability of scarce hospital beds.^18^,^20^

However, all aforementioned interventions are not yet integrated into the Dutch national guideline on EOS management, which was most recently revised in 2017.^21^ Some Dutch hospitals already adopted one or more of the antibiotic stewardship interventions independently, but they are not applied in the vast majority. To integrate novel evidence into daily practice, active implementation should take place that carefully considers determinants influencing its application, including perspectives of all stakeholders.^22 23^ One of the challenges in the transition from evidence to practice is that most clinical interventions are researched primarily in controlled settings, yielding type 1 and 2 evidence on disease aetiology, burden and intervention effectiveness. As a result, there is limited type 3 evidence on how these interventions function in real-world environments, making it unclear what should be targeted for effective implementation.^24^ Moreover, since interventions are usually studied individually, little is known about how they interact with other evidence-based approaches for the same disease. Regarding neonatal antibiotic stewardship, several studies have reported the effect of implementing antibiotic stewardship interventions, yet reported mainly the impact on antibiotic prescription rates rather than data on the implementation context and strategies.^25 26^ The few studies that did emphasise implementation strategies were conducted at neonatal intensive care units with Level IV facilities.^27 28^ In contrast, most Dutch late preterm and term neonates at increased risk of EOS are born and treated in secondary hospitals (Level I–II facilities) and receive care in collaboration with the obstetric department and primary neonatal care.

Therefore, the primary aim of this study is to assess the impact of the strategy to implement an antibiotic stewardship bundle (the EOS calculator, PCT-guided therapy and IV-to-oral switch therapy) on antibiotic exposure for EOS in Dutch secondary hospitals. Secondary aims are to assess additional clinical outcomes and implementation outcomes, including the acceptability, feasibility and fidelity of the interventions, as well as the appropriateness of the implementation strategies.

## Method

### Study design

**Figure 1:**
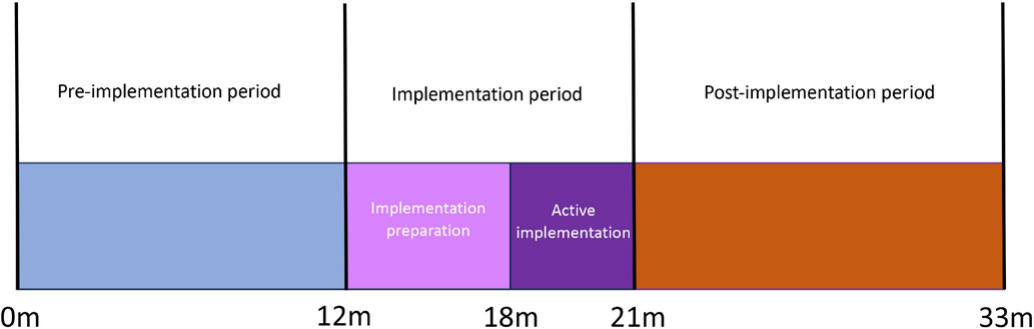
Implementation timeline.

This study is part of the Protect-Neo implementation project and focuses on the implementation and evaluation phase of the project. We will perform a multicentre, prospective, interrupted time series (ITS) and before-after implementation study. Mixed methods will be used to integrate clinical outcomes with implementation outcomes. The study runs from January 2024 to June 2026 and comprises a 9-month implementation period followed by a 12-month post implementation period during which data are collected. In addition, a 12-month pre-implementation period (January–December 2023) provides baseline data for the comparison of clinical outcomes (figure 1). The implementation period is divided into two distinct phases: a 6-month preparation phase (eg, change of local protocols, informing stakeholders, lobbying for equipment), followed by a 3-month active implementation phase (educational sessions, spread of informative materials, etc.).

### Study setting and population

The study is conducted at the paediatric or specialised neonatal departments of 11 Dutch secondary hospitals and their surrounding primary neonatal care networks, consisting of obstetric practices and maternity care services. In the Netherlands, neonates at risk for EOS are initially managed either in obstetric wards (Level I) or neonatal wards (Levels II–IV), depending on their age and clinical condition. Most late-preterm and term neonates receive care in secondary hospitals (Level I–II facilities). Following discharge, continuity of care is provided by midwives, maternity nurses and general practitioners. Midwives hold medical responsibility for the mother and clinically well-appearing neonates during the first 10 days postpartum, with support from maternity nurses in the home setting. In cases where neonates continue oral antibiotic therapy at home, medical responsibility remains with the paediatrician, while supported by primary care.

Participating sites were purposively sampled covering various Dutch regions to create a proper reflection of region-bounded differences in organisation of care. The study population is twofold: (1) neonates born at 34 weeks of gestation or later with suspected EOS that will receive care associated with the interventions and (2) paediatricians, paediatric residents, nurse practitioners, neonatal nurses, maternity nurses and parents who are the focus of the implementation strategies. Other involved professionals are pharmacists, microbiologists, midwives, gynaecologists and gynaecologic residents.

### Evidence-based antibiotic stewardship interventions

#### EOS calculator

The EOS calculator, which was developed by researchers of Kaiser Permanente, is applicable to neonates born at 34 weeks of gestation or later and can be used within the first 24 hours after birth.^10^ In this implementation project, it is advised to apply the EOS calculator to all neonates with increased risk of EOS, based on the presence of at least one risk factor or clinical symptom as specified in the current Dutch Society of Paediatrics’ guideline ‘Prevention and treatment of early onset neonatal infections’.^21^ The EOS calculator uses information on regional incidence and five maternal factors combined with the clinical evaluation of the neonate to assess individual EOS risk. The calculator then provides recommendations on the need for clinical observation using repeated evaluation of vital parameters, a blood culture and start of empirical intravenous antibiotics. In line with the protocol of the Dutch randomised controlled trial on EOS calculator safety and effectiveness, some deviations or clarifications have been made to the original EOS calculator’s approach in this study’s clinical protocol.^29^ First, the EOS calculator’s recommendation to obtain a blood culture and perform clinical observations is replaced with clinical observations only. This deviation reflects usual practices in the Netherlands, where obtaining a blood culture without subsequent antibiotic administration is uncommon. Second, evaluation of vital parameters is done every 3 hours instead of 4 hours, according to current Dutch workflows. Third, the EOS calculator gives no specific timeframe for observations in the ‘routine vitals’ recommendation, which is up to own interpretation. In this study’s clinical protocol, the duration has been standardised at 24 hours. For this implementation project, the EOS calculator is chosen over a 48-hour serial clinical examination approach, that also has the potential to significantly reduce antibiotic prescriptions, but demands more resources.^30^ The decision was based on the EOS calculator’s stronger evidence for reducing unnecessary antibiotic use, as well as logistical considerations regarding the demand for more staff and room capacity due to longer duration of the observation period of serial clinical examinations.

#### PCT-guided therapy

PCT-guided therapy applies to neonates born at 34 weeks of gestation or later, who at 12 hours after start of antibiotics (t=12) are classified as having low or medium risk of infection (Group 1) based on the presence of one or two abnormal findings among the following categories: risk factors, clinical symptoms and laboratory findings (online supplemental file 1, table S1). PCT levels are assessed at t=12 (±6 hours) and t=24 (±6 hours) with a minimum of 12 hours between the measurements, and if consecutive values fall within the reference interval of the age-adjusted nomogram (online supplemental file 1, figure S1), antibiotic therapy can be discontinued as early as the second PCT value is known. For neonates without two consecutive low values of PCT, for neonates that are classified as high risk (Group 2), and in case PCT is not available, the necessity of treatment is reassessed at t=36 based on clinical course and blood culture following current practice. In neonates with blood culture proven infection, treatment will always be continued for at least 7 days, targeting the pathogen.

#### IV-to-oral switch therapy

IV-to-oral switch therapy applies to neonates born at 34 weeks or later, with a birth weight of >2000 grams, who are still suspected of infection, despite a negative blood culture, based on clinical symptoms/risk factors and abnormal infection parameters at the discretion of the paediatrician. After 36 hours of intravenous antibiotic therapy, if the neonate tolerates oral (tube) feeding and is clinically well, treatment is switched to oral antibiotic therapy with amoxicillin suspension. The recommended daily dose will be according to the Dutch Paediatric Formulary, 60 mg/kg/day in two doses, based on recently published data.^31^ In case the neonate vomits within half an hour of ingestion, administration should be repeated. The first dose will always be administered in the hospital, to assess tolerance by the neonate and educate parents. Afterwards, the location of treatment will shift from the hospital to the home environment in case this is considered safe. The paediatrician will stay responsible for neonatal care during the entire treatment period; care will not formally be transitioned to primary care. Nevertheless, informing primary care professionals about antibiotic treatment of the neonate is an essential part of the discharge procedure, as they are often a first contact person for families in the maternity period.

### Implementation strategy

#### Implementation mapping

**Figure 2:**
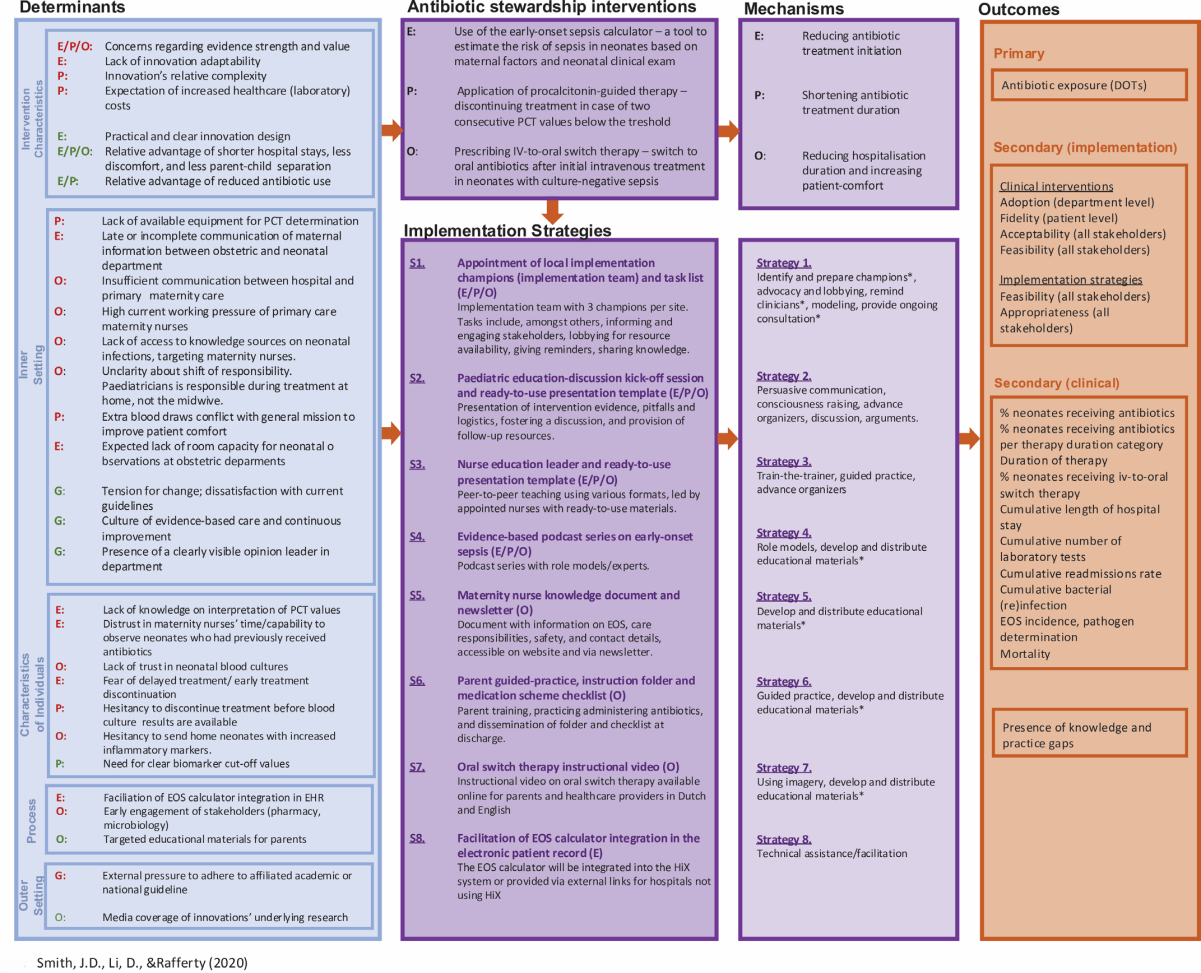
Implementation research logic model. Determinants are divided into barriers (red) and facilitators (green). DOTs, days on therapy; E, early-onset sepsis calculator; EHR, electronic health record; EOS, early-onset sepsis; IV, intravenous; O, IV-to-oral switch therapy; P, procalcitonin-guided therapy; PCT, procalcitonin.

**Figure 3:**
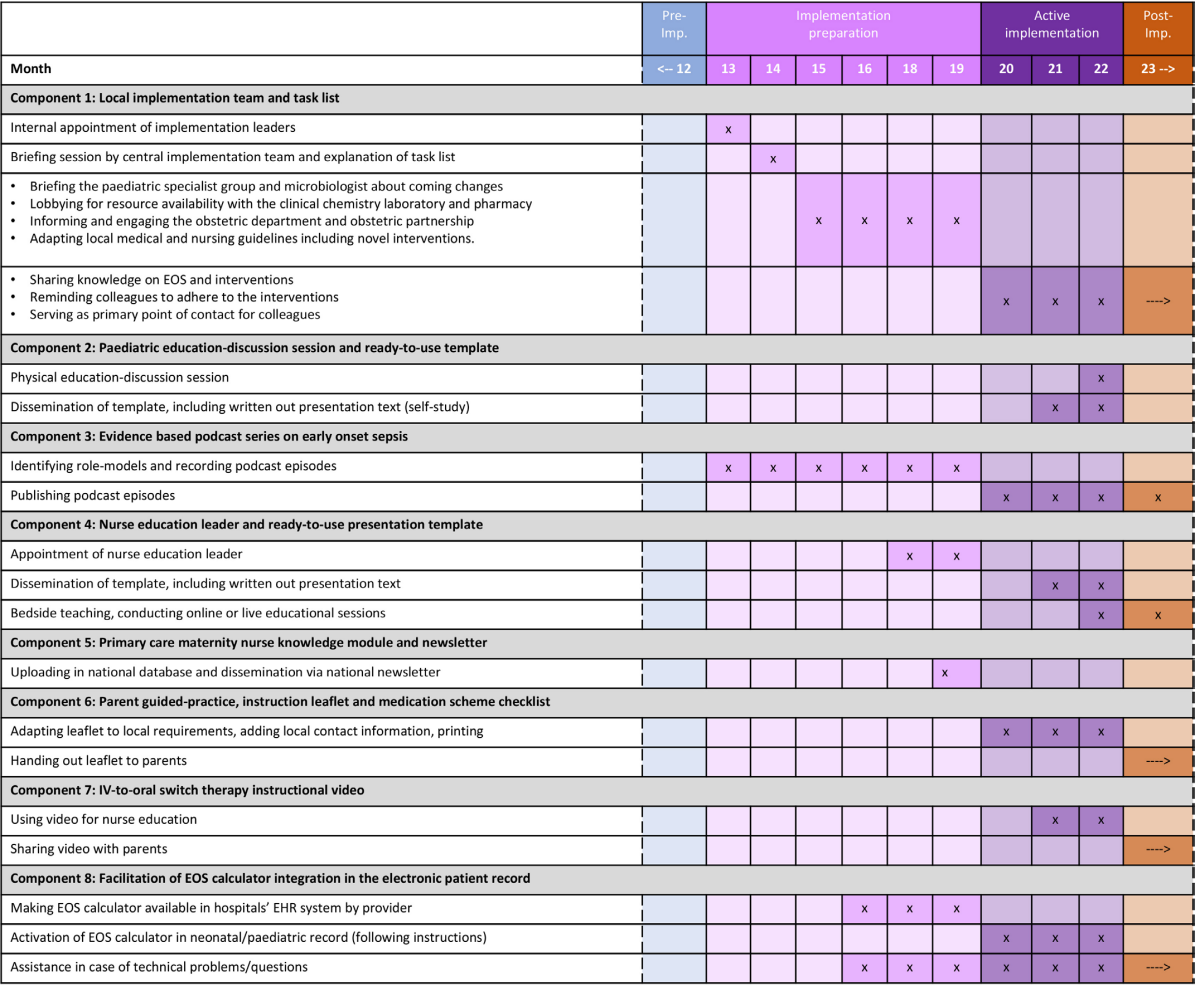
Implementation strategy components per study period. The symbol (**x**) indicates the specific time point at which a component is executed, while (→) represents the continuation of the practice beyond the active implementation period. EHR, electronic health record; EOS, early-onset sepsis.

We developed an implementation strategy, built of several sub-strategies, to facilitate implementation of the antibiotic stewardship interventions. The strategies were created by Implementation Mapping to design strategies with potential for successful real-world application. Implementation Mapping is distinct from intervention mapping and adapted to fit for design of implementation strategies.^32^ We combined the updated Consolidated Framework for Implementation Research to collect influencing determinants and the Taxonomy of Behavioural Change Methods to inform development of the strategies.^33 34^ All steps of the implementation mapping process are described in online supplemental file 2, tables S2–S4. Figure 2 gives a summary of determinants, implementation strategies, mechanisms and outcomes. Figure 3 gives an overview of the timeline per implementation strategy component.

### Implementation strategies

#### Strategy 1: Appointment of local implementation team and task list

Per site, an implementation team will be appointed, consisting of at least one nurse champion and two paediatrician champions (early adopters) that are tasked with leading implementation of the interventions within their hospital and its associated neonatal care network. The appointed implementation champions are respected individuals within the organisation who have the ability to motivate and engage their colleagues and have positive attitudes regarding the interventions. The implementation team will be briefed by the central study team and will be provided with a list of tasks and responsibilities. First, they will work towards their department’s adoption of the antibiotic stewardship interventions by (1) briefing and discussing the interventions with the paediatric specialist group and microbiologist, (2) reviewing resource availability with the clinical chemistry laboratory (PCT determination equipment) and pharmacy (amoxicillin suspension), (3) informing and engaging the obstetric department and obstetric partnerships and (4) adapting local medical and nursing guidelines to incorporate the interventions. Second, the implementation team supports implementation during the entire rollout of the project, by sharing their knowledge, reminding colleagues to adhere to the interventions and serving as primary point of contact for colleagues. To assist the local champions in discussing availability of resources and engaging stakeholders, the central research team provides tailored scientific information on PCT costs and IV-to-oral switch therapy, as well as a fact sheet with information about the intervention for the obstetric partnerships, if required. Moreover, the protocols of the antibiotic stewardship intervention will be made available in separate modules by the central research team, to facilitate integration of the interventions in the local protocol independently of each other.

#### Strategy 2: Paediatric education-discussion kick-off session and ready-to-use presentation template

Each local implementation team will organise a kick-off education-discussion session for the paediatric (resident) specialist team. The central research team facilitates a standardised presentation format to present data of current practice and room for improvement, followed by a concise overview of the evidence and practical application of the EOS calculator, PCT-guided therapy and IV-to-oral switch therapy. Subsequently, discussion will be encouraged by presenting potential pitfalls and actively asking for the participants’ perspectives. Afterwards, the presentation will be available for all paediatricians and paediatric residents, to be used for reference or reading in case of absence during the session. If required, the education discussion session can be repeated by the local implementation leader. The paediatricians and paediatric residents will be asked to sign off when they have attended the session, or gone through the information independently.

#### Strategy 3: Evidence-based podcast series on early onset sepsis

Five podcast episodes will be released, featuring experts and role models discussing essential topics regarding EOS. All scientific evidence discussed during the episodes will be linked in the show notes. The podcast is primarily focused on paediatricians and paediatric residents, but will also provide valuable information for microbiologists, pharmacologists, gynaecologists and neonatal nurses. The local implementation champions will actively send the link of each episode to their colleagues. The podcast will be shared on the publicly available free streaming Spotify, as well as the project’s website (http://www.neoinfecties.nl/).

#### Strategy 4: Nurse education leader and ready-to-use presentation template

Per site, one or more nurses will be appointed by the local implementation team as responsible for educating the nursing staff. These nurse education leaders will educate their peers on the antibiotic stewardship interventions through bedside teaching, conducting online or live educational sessions or providing an instructional email. A ready-to-use presentation will be available for the responsible nurses to use during education and to disseminate to all other nurses. All nurses are asked to sign off once they have received education on IV-to-oral switch therapy.

#### Strategy 5: Facilitation of EOS calculator integration in the electronic health record

In hospitals using the HiX (Chipsoft) electronic health record (EHR), the EOS calculator will be integrated directly into the patient record, eliminating the need to access it externally. The central research team will coordinate with Chipsoft to ensure the availability of the HiX calculator at participating sites and will provide an instruction manual for actually activating and using the calculator in practice. In hospitals not using HiX, a link to both the Kaiser Permanente website and the published open-source version of the EOS calculator will be provided, ensuring hospitals are not dependent on logistical, technical or maintenance issues associated with the Kaiser Permanente website.^35 36^

#### Strategy 6: Primary care maternity nurse knowledge module and newsletter

A targeted informative module on IV-to-oral switch therapy will be made available for all primary care maternity nurses on the National Knowledge Centre of Maternity Care’s website’s database (http://www.kckz.nl/), which is the formal website of Dutch maternity care guidelines. This module will cover general background information on EOS and IV-to-oral switch therapy, along with detailed information on care responsibilities, the instructions provided to parents regarding antibiotic administration, safety precautions and contact information. Additionally, this document, including further explanations, will be distributed to all maternity nurses through the KCZK’s email newsletter.

#### Strategy 7: Parent-guided practice, instruction leaflet and medication scheme checklist

Every parent taking a neonate home on oral antibiotic therapy will receive training from a neonatal nurse on antibiotics administration. After receiving instructions, parents will practise administering at least one dose in the hospital under the nurse’s guidance. In addition, a template for an instructional parent leaflet will be provided to all participating sites, allowing for adaptation to local communication needs and contact information. Parents will receive this instruction leaflet from the neonatal nurse at discharge. This includes detailed guidance on how to administer the antibiotics, steps to follow if a dose is missed or if the neonate vomits, warning signs and contact information for the hospital. The folder is available in both Dutch and English and written at a B1 language level. Additionally, a time schedule checklist is included, where the nurse or paediatrician will note the exact times and days for administering each dose at home. Parents are encouraged to use this checklist as a memory aid throughout the course of treatment.

#### Strategy 8: IV-to-oral switch therapy parental instructional video

An instructional video explaining oral antibiotic therapy, administration instructions, safety measurements and reasons to contact the neonatal department in case the neonate is at home will be made publicly available on a free streaming platform as well as the project’s website (http://www.neoinfecties.nl/), both in Dutch and English. This video primarily targets parents who go home with their neonate on oral antibiotics and is linked via a QR code on the parent instructional folder that every parent will receive at discharge. However, the video also serves as an educational source for neonatal nurses and maternity nurses and can support them in instructing parents.^34 35^

### Study outcomes

This study’s outcomes and their definitions have been streamlined as much as possible with those reported in literature, to pursue uniformity in data. Clinical outcomes are based on the Center for Disease Control and Prevention’s definitions for tracking antibiotic use and resistance and on the AENEAS (Analysis of Antibiotic Exposure and Early-Onset Neonatal Sepsis in Europe, North America, and Australia) study group’s proposed key indicators for reporting data on EOS and antibiotic use.^1 37^ Implementation outcomes are mainly based on Proctor’ taxonomy of implementation outcomes.^38^ All outcomes and their definitions are described in detail in online supplemental file 3, table S5.

#### Primary outcome

The primary outcome of this study is the total exposure to antibiotic therapy that was initiated within the first 72 hours after birth for the treatment of EOS. The exposure is measured in days on therapy (DOT), where a therapy day is defined as any calendar day when at least one dose of any antibiotic is administered. Besides the days on any antibiotic, also DOT per individual antibiotic agent (type and administration route) will be measured.

#### Secondary implementation outcomes—antibiotic stewardship interventions

We will determine the adoption of the three antibiotic stewardship interventions on department level. The fidelity to the antibiotic stewardship strategies will be determined on patient level, by calculating the proportion of eligible patients in which the intervention was applied. Professionals’ reported feasibility and acceptability of the interventions will be assessed to understand levels of adoption and fidelity and to inform possible future refinements of the interventions.

#### Secondary outcomes—process evaluation of multimodal implementation strategy

We will evaluate the process of implementation to analyse to what degree the implementation was successfully executed. Fidelity to the different implementation strategies will be measured, including detailed registration of potential ad hoc local adaptations of the strategy. The appropriateness of the different implementation strategies will be evaluated to determine their relevance and inform potential future improvement.

#### Secondary outcomes—clinical

We will determine the proportion of neonates starting on antibiotics per 100 live births, proportion of neonates receiving antibiotics per predefined category of duration (36 hours, 36–48 hours, 48–72 hours, >5 days and >7 days), duration of therapy, proportion of neonates receiving IV-to-oral switch therapy, duration of hospital stay, cumulative number of laboratory tests, cumulative readmission rate within 28 days after birth, cumulative bacterial (re)infection rate within 28 days after birth, incidence of culture-proven EOS per 1000 live births, pathogen determination and antibiotic susceptibility and infection-related mortality within 28 days after birth. For all (re)infections, we will provide exact data on timing of the infection within the first 28 days as well as the pathogen determination.

#### Secondary outcome—Paediatricians’ knowledge and practice gaps

We will assess the existence and potential change in paediatricians’ knowledge and practice gaps regarding EOS incidence, biomarker use and blood cultures.

### Data collection and data sources

To assess outcomes, quantitative and qualitative methods will be used. The main data sources are the EHRs, surveys, focus group evaluation sessions, an implementation logbook and field notes.

#### Data from the electronic health record

Data will be collected using automated data extraction methods of the hospitals’ business intelligence unit. Data will be sampled from all neonates with suspected EOS who were started on antibiotics during the pre- and post implementation period and include gestational age, birth weight, initiation of antibiotic treatment within the first 3 days of life, names and dosages of antibiotics used, duration of antibiotic treatment, blood culture results with pathogen identification, CRP and procalcitonin values within the first 28 days of life, readmissions within 28 days after discharge, re-initiation of antibiotic therapy within 28 days and discharge destination (home or transfer to another hospital). Additionally, the total number of live births during both periods will be obtained from hospital registries. All data will be securely stored in the Castor Electronic Data Capture (EDC) system.

#### Surveys

All paediatricians of the participating sites will be invited to complete a survey both before and 1 year after the active implementation period to assess changes in knowledge and practice gaps and the adoption of the interventions. Additionally, the post implementation survey will evaluate fidelity to and appropriateness of the implementation strategies 1–3 and 8 and assess acceptability and feasibility of the antibiotic stewardship interventions (onlinesupplemental files 4  5). Paediatric residents will only take part in the post implementation survey, as their frequent rotations across different workplaces make pre-implementation comparisons unfeasible. Neonatology nurses will also be invited to complete a survey, specifically to assess the acceptability and feasibility of IV-to-oral switch therapy and the fidelity to and appropriateness of strategies 1, 6 and 7 (online supplemental file 6). All surveys will be distributed via the Castor EDC system.

#### Focus group evaluation sessions

Focus group evaluation sessions with paediatricians, paediatric residents and nurse practitioners will be conducted at each site, 9–12 months after the implementation period was completed. The sessions will include 6–12 participants and have a duration of approximately 60 min. To ensure a sample that is as representative as possible, all focus groups will be conducted during regularly scheduled ward meetings during working hours, typically attended by all physicians on duty. These meetings naturally include a diverse group of individuals with varying roles and expertise within the department. Those expected to be on duty on the day of the focus group will be invited to participate by the local champions. Participants’ gender, professional role and years of working experience in their role will be recorded. The sessions will follow a semi-structured format, allowing the interviewer to explore qualitative insights into the implementation process and the antibiotic stewardship interventions (online supplemental file 7). All sessions will be audio-recorded, transcribed verbatim and securely stored on a departmental disk. Participant anonymity will be ensured by removing identifying information from the transcripts.

#### Semi-structured individual interviews

We will conduct parent interviews to evaluate the fidelity to and appropriateness of implementation strategies 6 and 7, as well as the acceptability and feasibility of IV-to-oral switch therapy (online supplemental file 8). Starting 3 months after the implementation begins, parents of each neonate discharged on oral therapy at participating sites will be invited to participate by their paediatricians. The first parent couple(s) per site who agree will be contacted by the interviewer to schedule the interview 1–4 weeks after discharge. Data on parents’ gender, age and number of children will be recorded. We will also conduct individual interviews with maternity nurses to evaluate the fidelity to and appropriateness of implementation strategies 1 and 5, as well as the acceptability and feasibility of IV-to-oral switch therapy (online supplemental file 9). Due to the relatively rare occurrence of neonatal oral therapy cases in the routine practice of maternity nurses, local obstetric partnerships and parents with a neonate on oral therapy will be asked to help identify maternity nurses who have been involved in the care for neonates with oral therapy at home. Data on maternity nurses’ gender and years of working experience will be recorded. For both maternity nurses and parents, we aim to conduct 12 interviews or as many as necessary to achieve data saturation. Data saturation is defined as the point at which no new topics emerge across three consecutive interviews. For all interviews, a semi-structured interview guide will be used. All sessions will be audio-recorded, transcribed verbatim and securely stored on a departmental disk. Participant anonymity will be ensured by removing identifying information from the transcripts.

#### Logbook

During the implementation phase and its preparation, the central research team will maintain a site-specific logbook to systematically document all implementation activities. This will include recording the completion dates of tasks assigned to local champions, verifying and updating local protocols and tracking the execution of implementation strategy components, such as distributing printed materials or uploading documents. Any ad hoc changes to the implementation strategies or adaptations to the interventions will also be logged. Data for the logbook will be collected from various sources, including site visits, email correspondence, phone calls and meetings with local implementation teams and other relevant stakeholders.

#### Field notes

During the implementation phase and its preparation, the central research team will maintain detailed field notes to document site-specific observations. These notes will capture information on implementation factors, how barriers were addressed and suggestions for future improvements. The primary sources of data for the field notes will include site visits, emails, phone calls, meetings with local implementation teams and interactions with other stakeholders. Data from the field notes will be systematically organised using a matrix based on the implementation strategies and antibiotic stewardship interventions.

### Analysis

#### Primary outcome

An ITS analysis will be conducted to compare trend of overall DOT (all types of antibiotic agents) per 1000 live births pre- and post-intervention. The ITS will have at least four data points in the pre-implementation period and four datapoints in the post implementation period, based on quarterly intervals. If the number of cases allows dividing the data into more datapoints, this may be considered. To account for potential clustering of observations within hospitals and potential differences between hospitals, we will apply a mixed-effects segmented regression analysis for ITS (‘joinpoint regression’) with DOT as dependent variable and random intercept for each hospital. Also, we will include random slopes for the post-intervention trend, allowing hospitals to differ not only in baseline DOT levels but also in how they respond to the implementation. Fixed effects include intervention phase (pre/post) and segmented time after the intervention to estimate both immediate level changes and changes in trend. Confounders, including the number of positive blood cultures, mean gestational age and mean birth weight will be included as time-varying covariates. Depending on data availability, we may also examine trends during the intermediate implementation phase to explore changes during this transition period as secondary analysis. Autocorrelation of residuals will be checked by using the Durbin-Watson test, and if present, corrected. Results will be reported as adjusted changes in level and trend, with 95%CIs, reflecting both pooled effects and between-hospital variability. Additionally, a before-after comparison will be performed using a t-test or Mann-Whitney U test, for both overall DOT and DOT per individual antibiotic agent.

#### Secondary outcomes

Quantitative data: Continuous data will be reported as mean with SD if normally distributed or as median with IQR if not normally distributed. Ordinal data, such as Likert scale responses, will be reported as medians with IQR. Other categorical data will be presented as frequencies with percentages with 95% CI where relevant. For comparisons between groups (pre-implementation and post implementation), continuous data will be analysed using an independent t-test if normally distributed or a Mann-Whitney U-test if not. Ordinal data will be compared using the Mann-Whitney U test, and other categorical data will be analysed using a χ^2^ test or Fisher’s exact test for small sample sizes. P values <0.05 will be considered statistically significant. All analyses will account for differences in group sizes where applicable.

Qualitative data: Transcripts of focus group evaluation sessions and interviews, field notes and open-ended survey questions will be analysed using a deductive analysis approach (directed content analysis). The first three evaluation sessions and interviews transcripts, as well as field notes and surveys, will be coded separately by two researchers, then compared and discussed to create consensus about codes. Subsequent transcripts, field notes and answers to open-ended questions will be coded by one researcher and discussed with the team in case of ambiguity. The data analysis will be an iterative process, with the analysis of the evaluation session or interview occurring before the following one.

For the implementation outcomes, data will be integrated following a convergent quantitative design, by combining quantitative data from the EHR, logbook and structured questionnaires with qualitative data from semi-structured interviews and field notes.^39^ This integration will allow us to gain a richer understanding of the adoption, fidelity, acceptability and feasibility of the antibiotic stewardship interventions, along with the fidelity to and appropriateness of the implementation strategies.

#### Sample size

Estimating the median DOTs in the Netherlands during the pre-implementation period is challenging due to the lack of data on the exact duration of antibiotic treatment for EOS in Dutch neonates. However, a mean antibiotic initiation rate of 3.8% of all live-born neonates was reported in 13 secondary Dutch hospitals.^40^ Additionally, another study among Dutch hospitals found that antibiotics were continued intravenously for more than 72 hours in 31.5% of cases in.^41^ Since neonates in the Netherlands are usually treated for either 24–72 hours (average 2 days) or 7 days, we can estimate the pre-implementation DOTs to be 136 per 1000 live-births (calculated as follows: (0.685×38 neonates×2 days) + (0.315×38 neonates×7 days)). This estimate matches the mean antibiotic exposure in other high-income countries, which is 135 (range 54–491) DOTs per 1000 live births as reported in the AENEAS study.^1^ Our goal for the post implementation period is to reduce the antibiotic initiation rate to 2% and halve the number of full treatment courses, aiming for 60 DOTs per 1000 live-births. However, we consider a reduction of at least 10% of DOTs to be clinically relevant. This would result in a post implementation outcome of 121.5 DOTs per 1000 live-births. Assuming a power of 90% and an alpha of 5%, a sample size of 2790 (1395 per group) would be required. This sample size will be feasible for both the total study population and most individual sites, since Dutch hospitals typically report annual birth rates ranging from 1000 to 3500, averaging approximately 2500 births per hospital per year. However, in hospitals with already low pre-implementation DOTs, no differences in absolute DOTS or trends may be found. This is acceptable, as this would demonstrate that these hospitals are already leading in the reduction of antibiotic use, and finding a difference through greater sample size would not be clinically relevant.

### Patient and public involvement

During the development of the study and the implementation strategies, parents’ perspectives were actively incorporated. Input was gathered through 11 parent interviews, ensuring their experiences and concerns were considered. Additionally, the neonatology parent organisation Care4Neo reviewed the original study plans, parent leaflet and instructional video, providing valuable feedback that led to revisions to better align with parents’ needs.

## Ethics and dissemination

The Medical Ethics Committee United (MEC-U) declared (reference: W24.132) that this study does not fall under the Dutch Medical Research Involving Human Subjects Act (WMO). Subsequently, ethical approval was granted by the Scientific Committee of the Franciscus Hospital (T110). The scientific committees of the following participating hospitals adopted this decision and granted permission for local conduct of the study: Albert Schweitzer Hospital Dordrecht, Noordwest Hospital Alkmaar, Antonius Hospital Sneek, Treant Hospital Emmen, Gelre Hospital Apeldoorn, Zuyderland Medical Centre Heerlen, Medical Centre Leeuwarden, Admiraal de Ruyter Hospital Goes, Tjongerschans Hospital Heerenveen, Catharina Hospital Eindhoven, Canisius Wilhelmina Hospital Nijmegen. As EHR data are sampled retrospectively and anonymously, a waiver of consent was given to collect these data. Informed consent will be obtained from participants completing surveys or taking part in interviews and focus group discussions. The findings will be disseminated through journal publications and conference presentations. Furthermore, practice and policy recommendations will be collaboratively developed with partner organisations.

## Supplementary material

10.1136/bmjopen-2025-103368 online supplemental file 1

10.1136/bmjopen-2025-103368 online supplemental file 2

10.1136/bmjopen-2025-103368 online supplemental file 3

10.1136/bmjopen-2025-103368 online supplemental file 4

10.1136/bmjopen-2025-103368 online supplemental file 5

10.1136/bmjopen-2025-103368 online supplemental file 6

10.1136/bmjopen-2025-103368 online supplemental file 7

10.1136/bmjopen-2025-103368 online supplemental file 8

10.1136/bmjopen-2025-103368 online supplemental file 9
