# Supplementary material for: Implementation strategy for an antibiotic stewardship bundle to promote optimal treatment choices in neonates with suspected early-onset sepsis (Protect-Neo): a study protocol for a multicentre, prospective interrupted time series and before-after study
Source: BMJ Open. 2025 Nov 4;15(11):e103368. doi: 10.1136/bmjopen-2025-103368 (PMC12588035; doi:10.1136/bmjopen-2025-103368)
Supplement: online supplemental file 1 [file bmjopen-15-11-s001.docx]

**Supplementary file 1 -** Additional information for PCT-guided therapy


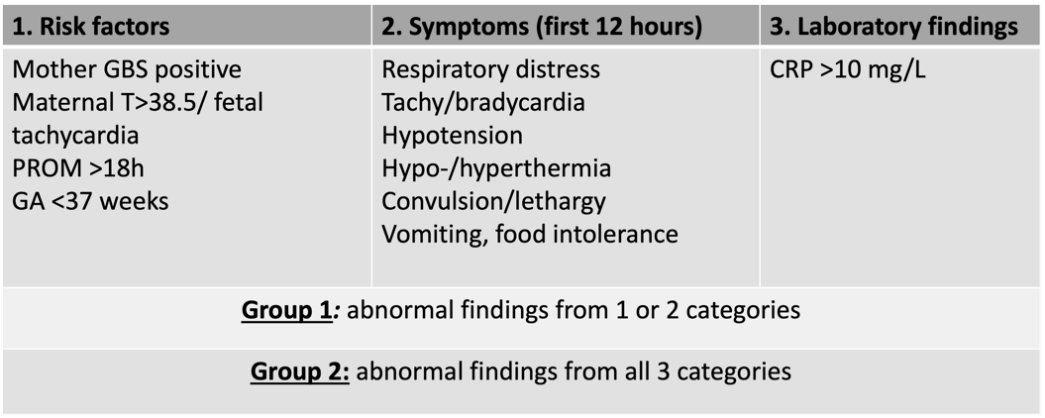


**Table S1** Risk classification of neonates started with antibiotic therapy for suspected early-onset sepsis. Group 1=low-intermediate risk, Group 2=high risk. PCT guided therapy is only applicable for neonates in Group 1.


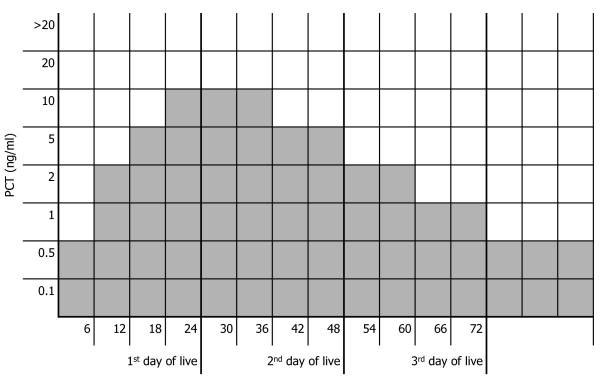


**Figure S1** Age-adjusted nomogram of procalcitonin (PCT) values in the first 72 hours of life
