# Supplementary material for: Implementation strategy for an antibiotic stewardship bundle to promote optimal treatment choices in neonates with suspected early-onset sepsis (Protect-Neo): a study protocol for a multicentre, prospective interrupted time series and before-after study
Source: BMJ Open. 2025 Nov 4;15(11):e103368. doi: 10.1136/bmjopen-2025-103368 (PMC12588035; doi:10.1136/bmjopen-2025-103368)
Supplement: online supplemental file 2 [file bmjopen-15-11-s002.docx]

**Supplemental file 2**

**Implementation mapping**

Step 1: Identify adopters, implementers, stakeholders, and assess their implementation needs.

In this first step we conducted a thorough assessment to identify all stakeholders, with a specific focus on adopters and implementers, and to determine the needs, barriers, and facilitators relevant to implementing the interventions. Stakeholders were identified through a detailed mapping of the neonatal infection care workflow, supplemented with insights from professionals actively working in the field. The main adopter of all three antibiotic stewardship interventions was identified as the paediatric specialist group, that jointly decides to adopt novel policy recommendations, depending on resources provided by the medical chemistry laboratory and pharmacy. Paediatricians and paediatric residents were identified as the primary implementers for all three interventions and additionally neonatal nurses, parents and primary care maternity nurses for IV-to-oral switch therapy. Other stakeholders for IV-to-oral switch therapy are microbiologists, pharmacists and primary care midwives. Other stakeholders for the EOS calculator are gynaecologists, obstetricians, and gynaecologic residents. Interdisciplinary focus groups were conducted to assess contextual determinants that are related to implementing the interventions[1]. Determinants were coded using the 49 CIFR constructs, categorized in five domains: the intervention, outer setting, inner setting, individuals and implementation process [2].

Step 2: Defining outcomes, performance objectives, determinants and create matrices of change.

Based on insights from step 1, we defined specific adoption and implementation objectives with underlying performance objectives per adopter or implementer (Table S2). Performance objectives were formulated based on the needs that were identified during step one. To facilitate the selection of evidence-based strategies based on the Taxonomy of Behaviour Change Methods (TBCM), the emerged CFIR constructs during step 1 were translated into the relevant determinant groups of the TBCM [3]: (1) Knowledge, (2) Awareness and Risk perception, (3) Attitude, Beliefs and Outcome Expectations, (4) Skills, Capability, Self-efficacy, (5) Habitual Behaviour, (6) Evironmental Conditions. Then, performance objectives were linked to the identified determinants to create matrices of change, resulting in concrete change objectives (Table S3).

Step 3: Selecting theoretical methods and design implementation strategies

In step 3, we chose theory-based strategies that linked to the determinants identified in Step 1 and 2. Strategies were mainly selected using the Taxonomy of Behaviour Change Methods, supplemented by some strategies that were developed during the Expert Recommendations for Implementing Change (ERIC) project [4]. The strategies selected targeted both individual-level factors, such as enhancing knowledge and skills, and organizational-level factors, like the availability of resources. Practical applications of the theoretical strategies were designed to operationalize these methods in ways that fit the local context. Eight main components of the strategy can be distinguished, which consist of several sub-strategies. The detailed description of underlying evidence-based strategies, target group and practical application can be found in Table s4.

**Table S2.** Roles, implementation outcomes and performance objectives.

| **Role** | **Implementation outcomes** | **Performance objectives**  *B = Bundle of interventions, E = EOS calculator specific. P = PCT guided therapy specific*  *O = Oral switch therapy specific* |
| --- | --- | --- |
| **Paediatrics specialist team** (adopter of the EOS calculator, PCT-guided therapy and Oral switch therapy)  Involved stakeholders: Clinical chemistry laboratory, Microbiology department, hospital’s pharmacy | The paediatric specialist team, or their representatives, at the hospital decides to adopt the EOS calculator, PCT-guided therapy and oral switch therapy, by integrating these interventions into their formal local EOS management protocol. | B1. Compare the bundle of antibiotic stewardship interventions to current practices  B2. Gain support from stakeholders  P1. Discuss availability of PCT determination equipment with clinical chemistry department  O1. Discuss availability of a neonate-suited amoxicillin suspension with the hospital’s pharmacy  B3. Agree to implement the bundle of antibiotic stewardship interventions  B4. Agree to change the local nursing and medical protocols integrating the antibiotic stewardship interventions as intended by the program developers.  B5. Provide a program champion |
|  | The paediatric specialist team at the hospital integrates the EOS calculator, PCT-guided therapy and oral switch therapy into their formal local protocol exactly as prescribed by the program developers. |  |
| **Paediatricians, paediatric residents** (EOS calculator, PCT-guided therapy and IV-to-oral switch therapy) | Paediatricians and residents decide apply the adapted local protocol (integrating the EOS calculator, PCT-guided therapy, oral switch therapy | B1. Look up the adapted guideline in the hospital’s database  B2. Compare the adapted guideline with the current guideline  B3. Learn evidence and practical application of the interventions  B4. Choose the novel interventions over current practices |
|  | Paediatricians and residents apply the EOS calculator, PCT-guided therapy and oral switch therapy to eligible neonates as intended by the program developers. | E1. Fill in the EOS calculator in all eligible neonates (1 or more risk factors, or clinical symptoms related to EOS)  E2. Follow the policy recommendation of the EOS calculator (24 hours observation, or drawing a blood culture and starting antibiotics)  P1. Correctly interpret the PCT nomogram  P2. Order PCT tests at the right time in eligible neonates (low and medium risk)  P3. Stop therapy in case of two consecutive low PCT values and a negative blood culture.  O1. Think of switching to oral antibiotics in all eligible neonates (negative culture, increased inflammatory values, clinically well)  O2. Look up the correct dose of oral switch therapy |
|  | Paediatricians and residents have increased knowledge on relevant topics with regard to early-onset sepsis | B1. Learn the evidence-based role of biomarkers in EOS management and the correct collection and interpretation of blood cultures |
| **Neonatology nurses** ((IV-to-oral switch therapy) | Nurses provide parents, that go home with a neonate on oral antibiotics, with relevant instructions | B1. Look up the novel oral switch therapy guideline in the hospital’s database  B2. Compare the guideline with current practices  B3. Gather knowledge on practical application of oral switch therapy  B4. Choose to apply the novel tasks |
|  | Nurses provide the parent instructions on oral antibiotic therapy at home to alle eligible parents as intended by the program developers. | O1. Instruct parents to collect the antibiotic suspension at the pharmacy  O2. Demonstrate to parents how to administer the antibiotic suspension to the neonate  O3. Provide parents with safety instructions and reasons to contact the hospital |
| **Parents/caregivers** (IV-to-oral switch therapy) | Parents will administer the oral antibiotic suspension to their neonate in the right dose, at the right time and take safety measurements and call the hospital when indicated. | O1. Collect the correct equipment to administer oral antibiotic suspension  O2. Learn about correct oral antibiotic administration, safety measurements and reasons to contact the hospital  O3. Look up information on safety measurements, reasons to contact the hospital and the phone number of the paediatric department when indicated |
| **Gynaecologists/obstetricians, gynaecologic residents** (EOS calculator) | Deliver timely and complete information on maternal risk factors to the paediatrician or resident in order to fill in the EOS calculator | E1. Learn the evidence and practical application of the EOS calculator, including the consequences of missing information  E2. Keep track of relevant maternal risk factors, including gestational age, maternal temperature, time of rupture of membranes, GBS status, and antibiotics use. |
| **Primary care maternity nurses, primary care midwives** (IV-to-oral switch therapy) | Maternity nurses and midwives correctly answer or redirect parents’ questions regarding oral antibiotics or neonatal infection when the neonate is treated at home. | O1. Receive a discharge letter on the medical condition of the newborn, with neonate specific instructions.  O2. Have access to information on antibiotic administration, safety criteria, or reasons for parents to contact the hospital  O3. Express self-confidence to accommodate and guide parents who come home with a neonate on oral antibiotics |
| **Microbiologist** (IV-to-oral switch therapy) | The microbiologist advises paediatricians to switch from intravenous to oral antibiotics in eligible neonates | O1. Compare oral switch therapy to current practices  O2. Change the local antibiotic guidance protocol |

**Table S3***.* Matrices of change

| **Performance objective (B/E/P/O)**  *B = Bundle of interventions*  *E = EOS calculator specific*  *P = PCT guided therapy specific*  *O = Oral switch therapy specific* | **Knowledge (K)**  *(CIFR individual characteristics domain)* | **Risk perception and awareness (R)**  *(CFIR inner setting and individual characteristics domain* | **Attitude, beliefs, outcome expectations (A)**  *(CFIR intervention, inner setting, and individual characteristics domain)* | **Skills, capability, self-efficacy (S)**  *(CFIR individual characteristics)* | **Habitual behaviour (H)**  *(CFIR individual characteristics domain)* | **Environmental factors (E)**  *(CFIR outer setting, inner setting, implementation process domain)* |
| --- | --- | --- | --- | --- | --- | --- |
| **Representatives of the paediatric specialist group (adopter)** | | | | | | |
|  | | | | | | |
| **B1. Compare the bundle of antibiotic stewardship interventions to current practices** | **B1.K1.** Describe the most important evidence behind the EOS calculator, PCT guided therapy, oral switch therapy, and the current guideline  **B1.K2**. Describe the policy recommendations of the EOS calculator, PCT-guided therapy, oral switch therapy, and the current guideline. |  | **B1.A1**. Are open to consider novel interventions for clinical practice | **B1.S1**. Are able to critically review and compare scientific evidence |  | **B1. E1.** Have easy access to the adapted guideline  **B1.E2**. Have access to tailored information and evidence on the antibiotic stewardship interventions |
| **B2. Gain support from stakeholders** | **B2.K1**. Describe key point to discuss with stakeholders with regard to the antibiotic stewardship interventions | **B2. R1.** Are aware that besides themselves, more stakeholders are involved in the implementation of the antibiotic stewardship interventions | **B2. A1.** Express the belief that support from stakeholders is essential for proper implementation  **B2.A2.** Express the belief that stakeholder engagement will improve their cooperation | **B2. S1.** Express confidence in their ability to engage stakeholders |  |  |
| **P1. Ensure availability of PCT determination equipment.** | **P1.K1**. Describe the most relevant evidence for PCT-guided therapy  **P1.K2.** Describe the equipment needed for determination of PCT values  **P1.K3**. Describe the added value of PCT testing compared to usual CRP testing | **P1.R1**. Are aware that PCT determination equipment might not be available in their hospital | **P1.A1**. Expect that PCT-testing will improve current neonatal care  **P1.A2** Expect that PCT-testing will not lead to increased costs | **P1.S1.** Possess techniques to convey the importance of PCT testing to the clinical chemistry laboratory |  |  |
| **O1. Ensure the availability of an amoxicillin suspension suitable for neonates** | **O1.K1**. Describe the type, dose and formula of antibiotics needed for oral switch therapy  **O1.K2.** Describe the most relevant evidence for oral switch therapy | **O1.R1**. Are aware that a neonate-suited amoxicillin suspension might not be available in the hospital’s pharmacy | **O1.A1.** Expect that oral switch therapy will improve current neonatal care | **O1.S1**. Possess techniques to convey the importance of oral switch therapy to the pharmacists |  |  |
| **B3. Agree to implement the bundle of antibiotic stewardship interventions** | **B3.K1**. Describe the steps needed to implement the antibiotic stewardship interventions  **B3.K2**. Describe the barriers that have to be overcome to implement the antibiotic stewardship interventions | **B3.R1.** Are aware of the short- and long-term risks of antibiotic use in the early neonatal period.  **B3.R2**. Are aware of the number of neonates currently being treated for suspected EOS and the number of neonates with culture-proven sepsis.  **B3.R3.** Are aware of the low adherence to the current guideline and the large differences in clinical practice between different hospitals and paediatricians | **B3.A1**. Expect that the EOS calculator will safely decrease antibiotic therapy rates  **B3.A2.** Expect that PCT-guided therapy will decrease antibiotic therapy duration  **B3.A3**. Expect that oral switch therapy will result in shorter hospital stay and increased patient comfort  **B3.A4**. Expect that oral switch therapy will not result in increased antibiotic prescriptions | **B3.S1.** Express confidence in the neonatal care network to arrange the workflows  **B3.S1.** Describe the hospital and their surrounding neonatal care network capable and ready of change |  | **B3.E1**. Equipment for PCT determination is available, or resources are available to buy novel equipment.  **B3.E2**. Amoxicillin suspension suited for neonates is available in the hospital’s pharmacy |
| **B4. Agree to change the local nursing and medical protocols integrating the antibiotic stewardship interventions as intended by the program developers.** | **B4.K1**. Describe how the EOS calculator, PCT-guided therapy and oral switch therapy were carried out in the most relevant clinical studies | **B4.R1.** Are aware of the lack of evidence on observing neonates less than 24 hours in case of low infection risk according to the EOS calculator  **B4.R2** Are aware that PCT-guided therapy is only suited for neonates with low or medium infection risk | **B4.A1.** Believe it is safe to terminate antibiotics in case of two consecutive PCT values, before blood culture results are known  **B4.A2.** Believe it is safe to switch to oral antibiotics in eligible neonates after 36 hours of IV therapy  **B4.A3**.Believe there is enough room capacity to observe neonates at the maternity ward for a longer period of time |  | **B4.H1** Overcome the habit to observe neonates considered as low risk for only 12 hours | **B4.E1.** Expected available room capacity at the maternity ward to observe neonates  **B4.E2** Agreement of obstetrics department to increase observation duration. |
| **B5. Provide a program champion** | **B5.K1.** Describe the role and tasks of a program champion |  | **B4.A4.**Believe the program champion is an important part of implementation  **B4.B5.** Expect the program champion will enable smooth implementation and maintenance of the antibiotic stewardship interventions. | **B4.S1**. Express confidence in the ability to recruit a program champion. |  |  |
| **Paediatricians, paediatric residents (implementer)** | | | | | | |
|  | | | | | | |
| **B1. Look up the adapted guideline in the hospital’s database** | **B1.K1**. Describe were to find the adapted guideline |  |  |  |  | **B1.E1**. Have access to the adapted guideline |
| **B2. Compare the adapted guideline with the current guideline** | **B2.K1**. Describe the most important evidence behind the EOS calculator, PCT guided therapy, oral switch therapy, and the current guideline  **B1.K2**. Describe the policy recommendations of the EOS calculator, PCT-guided therapy, oral switch therapy, and the current guideline. |  | **B2.A1**. Are open to consider novel interventions for clinical practice | **B2.S1**. Express confidence in the ability to critically review and compare scientific evidence |  |  |
| **B3. Learn the evidence and practical application of the interventions** | **B3.K1.** Describe where to find educational materials on EOS calculator, PCT guided therapy, and oral switch therapy. | **B3.R1.** Recognize that education is essential for the effective implementation of the interventions. | **B3.A1.** Express a commitment to investing in learning to enhance patient care. |  | **B3.H1.** Are willing to change their current EOS related policy choices | **B3.E1.** Have access to tailored educational materials  **B3.E2.** Have time available to attend educational meetings or read/listen/watch educational materials. |
| **B4. Choose the apply novel interventions over current practices** |  | **B4.R1**. Are aware of the short- and long-term risks of antibiotic use in the early neonatal period.  **B4.R2**. Are aware of the number of neonates currently being treated for suspected EOS and the number of neonates with culture-proven sepsis.  **B4.R3**. Are aware of the low adherence to the current guideline and the large differences in clinical practice between different hospitals and paediatricians | **B4.A1.** Express a commitment to prioritizing patient-centered care.  **B4.A2.** Believe that the EOS calculator will not lead to missed cases of EOS.  **B4.A3.** Have confidence that the EOS calculator will effectively reduce antibiotic use.  **B4.A4.** Believe that the improved patient comfort from a shorter duration of antibiotic therapy outweighs any discomfort caused by additional blood draws. | **B4.S1.** Express confidence in the ability to fill in the the EOS calculator.  **B4.S2.** Express confidence in the ability to interpret EOS calculator recommendations.  **B4.S3.** Express confidence in the ability to correctly interpret PCT values.  **B4.S4.** Express confidence in the ability to prescribe oral therapy for a neonate. | **B4.H1.** Resist the tendency to stick to the familiar practice of choosing to start antibiotics based on risk categorization.  **B4.H2**. Discontinue the use of CRP as a biomarker for guiding early-onset sepsis (EOS) management.  **B4.H3.** Refrain from automatically prescribing intravenous therapy for cases of culture-negative sepsis. | **B4.E1.** Have access to the EOS calculator  **B4.E2.** Have access to the PCT nomogram  **B4.E3.** Availability of PCT determination equipment  **B4.E4.** Availability of oral amoxicillin suspension |
| **E1. Fill in the EOS calculator in all eligible neonates (1 or more risk factors, or clinical symptoms related to EOS)** | **E1.K1.** Describe the target group for EOS calculator use  **E1.K2.** Describe risk factors and clinical symptoms associated with oral switch therapy | **E1.R1.** Are aware of the influence of risk factors/clinical symptoms on EOS risk | **E1.A1.** Belief the EOS calculator is of added value in all eligible neonates |  | **E1.H1.** Resist the tendency to stick to the familiar practice of choosing to start antibiotics based on risk categorization. |  |
| **E2. Follow the policy recommendation of the EOS calculator (24 hours observation, or drawing a blood culture and starting antibiotics)** | **E2.K1.** Describe the optional policy recommendations of the EOS calculator, and their practical application | **E2.R1.** Are aware of the lack of evidence on observing neonates less than 24 hours in case of low infection risk following the EOS calculator | **E2.A1.** Belief that it is necessary for safety to observe neonates with low risk for at least 24 hours |  |  | **E2.E1.** Actual available room capacity at the maternity ward to observe neonates  **E2.E2.** Agreement of the gynaecologist/obstetrician, or gynaecologic resident to observe for 24 hours |
| **P1. Correctly interpret the PCT nomogram** | **P1.K1.** Describe the physiological factors that influence PCT values  **P1.K2**. Describe when PCT levels begin to rise and reach their peak in the blood. |  |  | **P1.S4.** Feel capable of correctly interpret the PCT values |  | **P1.E1.** Have access to the PCT nomogram |
| **P2. Order PCT tests at the right time in eligible neonates (low and medium risk)** | **P2.K1.** Describe the indications for PCT- guided therapy | **P2.R1.** Are aware that PCT-guided therapy is only investigated in neonates with low or medium infection risk | **P2.A1.** Belief that PCT-guided therapy should only be applied to neonates with low or medium infection risk  **P2.A2.** Express the willingness to use the NeoPIns algorithm, to decide if a neonate is suited for PCT-guided therapy. | **P2.S1.** Express self-consciousness to differentiate neonates with low-medium infection risk to neonates with high infection risk. |  |  |
| **P3. Stop therapy in case of two consecutive low PCT values and a negative blood culture.** | **P3.K1.** Describe the conditions in which therapy can be stopped when using PCT-guided therapy |  | **P3.A1.** Belief it is safe to discontinue therapy based on two low PCT values before blood culture results are known | **P3.S1.** Feel capable of correctly interpreting the PCT values | **P3.H1.** Resist the tendency to stick to the familiar practice of always waiting on blood culture results to make further policy choices. |  |
| **O1. Think of switching to oral antibiotics in all eligible neonates (negative culture, increased inflammatory values, clinically well)** | **O1.K1.** Describe the eligibility criteria for prescribing oral switch therapy |  |  | **O1.S1.** Express self-consciousness to identify neonates that are eligible for oral switch therapy |  |  |
| **O2. Look up the correct dose of oral switch therapy** | **O2.K1**. Describe the location of information sources for dosing schedules of oral switch therapy | **O2.R1**. Are aware that the daily dosage of amoxicillin in case of oral switch therapy is lower than current dosages. |  |  | **O2.H1.** Discontinue prescribing amoxicillin at the usual, higher dosage. | **O2.E1.** Have online access to information on oral switch therapy dosing schemes |
| **A5. Increase their knowledge on the role of biomarkers in EOS management and the correct collection and interpretation of blood cultures** | **B5.K1**. Describe that biomarkers (CRP, PCT) do have low positive predictive value for EOS, and should therefore not be used to decide to start or continue treatment  **B5.K2**. Describe that biomarkers (CRP, PCT) do have good negative predictive value for EOS, and can be used to decide to safely discontinue treatment  **B5.K3**. Describe that the minimum amount of blood volume for a proper neonatal blood culture is 1 mL  **B5.K4.** Describe that properly collected blood cultures have high sensitivity for EOS  **B5.K5.** Describe that blood cultures should be stored at room temperature if they are not directly processed. | **B5.R1.** Are aware that their current thinking patterns with regard to biomarker interpretation may not be correct  **B5.R2**. Are aware that their current thinking patterns with regard to biomarker collection interpretation may not be correct | **B5.A1.** Are open to revise current thinking patterns  **B5.A2**. Are willing to put in the effort to gather additional knowledge |  | **B5.H1**. Resist the tendency to fall back into old thinking patterns | **B5.E1.** Have access to tailored educational sources  **B5.E2.** Have time available to attend educational meetings or read/listen/watch educational materials. |
| **Neonatology nurses** | | | | | | |
|  | | | | | | |
| **A1. Look up the novel oral switch therapy guideline in the hospital’s database** | **B1.K1.** Describe where to find the hospital’s guidelines |  |  |  |  |  |
| **A2. Compare the guideline with current practices** | **B2.K1.** Describe the policy recommendations of both oral switch therapy and current practices, and the differences between their application in daily clinical practice |  | **B2.A2.** Are open to consider oral switch therapy as novel clinical practice | **B2.S1.** Express confidence in the ability to review and compare two guidelines |  | **B2.E1.** Have access to the current guideline (if available)  **B2.E2.** Have access to the novel/adapted guideline |
| **A3. Learn the practical application of oral switch therapy** | **B3.K1.** Describe where to find educational materials on oral switch therapy | **B3.R2.** Recognize that education is essential for the effective implementation of oral switch therapy | **B3.A3.** Express a commitment to investing in learning to enhance patient care. |  |  | **B3.E1.** Have access to tailored educational materials  **B3.E2.** Have time available to attend educational meetings or read /listen/watch educational materials |
| **O1. Instruct parents to collect the antibiotic suspension at the pharmacy before discharge** | **O1.K1.** Describe the steps needed for parents to collect antibiotics at the pharmacy | **O1.R1.** Are aware of the reasons that parents have to collect the antibiotics before discharge |  | **O1.S1.** Possess the skills to provide parents with instructions tailored to their needs and educational level.  **O2.S1**. Express confidence in the ability to provide clear parental instructions |  | **O1-3.E1.** Possess materials to support parent instructions.  **O1-3.E2**. Have access to an interpreter phone |
| **O2. Demonstrate to parents how to administer the antibiotic suspension to the neonate** | **O2.K1.** Describe the steps needed for administration of oral therapy |  | **O2.A3.** Express the belief that parents are able to administer antibiotics to their newborn home |  |  |  |
| **O3. Provide parents with safety instructions and reasons to contact the hospital** | **O3.K1.** Describe that oral antibiotics should be readministered if a neonate vomits within half an hour after administration  **O3.K2.** Describe that oral antibiotics cannot be mixed with milk  **O3.K3.** Describe reasons to contact the hospital | **O3.R1.** Are aware of the risk missing a gift of antibiotic therapy  **O3.R2.** Are aware of the risk of mixing antibiotics with milk | **O3.A3.** Express the belief that parents are able to take safety measurements and call the hospital when indicated. |  |  |  |
| **Parents/caregivers** | | | | | | |
|  | | | | | | |
| **O1. Collect the correct equipment to administer oral antibiotic suspension** | **O1.K1.** Describe which equipment is needed for antibiotics administration  **O1.K2.** Describe where to collect the amoxicillin suspension  **O1.K3.** Describe where to collect the syringe for administration | **O1. R1.** Are aware the medication and syringe should be collected in order to administer oral antibiotic therapy |  |  |  | **O1.E1**. Availability of oral amoxicillin suspension  **O1.E2.** Availability of syringes for administration  **O1.E3.** Not having to pay for collecting the medication |
| **O2. Learn about correct oral antibiotic administration, safety measurements and reasons to contact the hospital** | **O2.K1.** Know the methods to administer oral antibiotics  Describe the correct dose of antibiotics  **O2.K2.** Describe the correct frequency and duration of antibiotics administration  **O2.K3**. Describe what to do in case the neonate vomits  **O2.K4.** Describe what to do in case of a missed dosage  **O2.K5.** Describe reasons to contact the hospital | **O2.R1.** Are aware of the risks of not correctly or timely administering antibiotics to their neonate  **O2.R2.** Are aware of the signs that suggest recurrent infection in their newborn | **O2.A1.** Belief it is important to correctly and timely administer antibiotics to their neonate  **O2.A2.** Belief the need of proper infection treatment outweighs the possible side effects of antibiotics  **O2.A3.** Feel no barrier to calling the hospital | **O2.S1.** Express confidence in their skills to administer antibiotics at home to their neonate  **O2.S2.** Express confidence in their self-efficacy to administer antibiotics at home to their neonate  **O2.S3.** Express confidence in their ability to recognize alarm symptoms. |  | **O2.E1**. Have access to instructions and training in the hospital |
| **O3. Look up information on safety measurements, reasons to contact the hospital and the phone number of the paediatric department when indicated** | **O3.K1**. Know where to find informational materials with instructions on antibiotics administration, safety measurements, and reasons to contact the hospital | **O3.R1.** Are aware of the existence of informational materials |  | **O3.S1.** Express confidence in their ability to find the informational materials  **O3.S2**. Express confidence in their ability to understand the informational materials |  | **O3.E2.** Have access to clear and easy-to-understand informational material. |
| **Gynaecologists/obstetricians, gynaecologic residents** | | | | | | |
|  | | | | | | |
| **E1. Learn the evidence and practical application of the EOS calculator** | **E1.K1.** Describe where to find educational materials on the EOS calculator | **E1.R1.** Recognize that education is essential for the effective implementation of the interventions. | **E1.A1.** Express a commitment to investing in learning to enhance patient care. |  | **E1.H1**. Are willing to change their current EOS related policy choices | **E1.E1.** Have access to tailored educational materials  **E1.E2.** Have time available to attend educational meetings or read/listen/watch educational materials. |
| **E2. Keep track of relevant maternal risk factors, including gestational age, maternal temperature, time of rupture of membranes, GBS status, and antibiotics use.** | **E2.K1.** Describe the maternal risk factors need to be known for EOS calculator application  **E2.K2.** Describe why the maternal risk factors need to be known for EOS calculator application | **E2.R1.** Are aware of the impact of missing information on the inability to use the EOS calculator | **E2.A1.** Express the willingness the support improvement of neonatal care  **E2.A2.** Belief the EOS calculator will reduce unnecessary antibiotics use in neonates  **E2.A3.** Belief all maternal risk factors are of relevance for EOS calculator application | **E2.S1.** Express confidence in their ability to keep track of information on relevant maternal risk factors |  |  |
| **Primary care maternity nurses, primary care midwives** | | | | | | |
|  | | | | | | |
| **O1. Articulate the agreements on responsibilities in the care of neonates coming home with oral antibiotics** | **O1.K1.** Describe that the paediatrician is responsible for treatment of the neonate during oral switch therapy  **O1.K2**. Describe that parents can directly call the paediatric department, without referral by the midwive or general practitioner. | **O1.R1.** Are aware of altered neonatal care responsibilities in case of oral switch therapy | **O1.A1**. Belief that the hospital will accept parents’ calls, without referral by the midwive or general practitioner |  |  | **O1.E1.** Have access to information sources, describing responsibility agreements |
| **O2. Learn the reason for antibiotic administration at home, safety criteria, and reasons for parents to contact the hospital** | **O2.K1.** Describe where to find educational materials on oral switch therapy | **O2.R1**. Recognize that education is essential for the effective implementation of oral switch therapy | **O2.A1.** Express a commitment to investing in learning to enhance patient care. | **O2.S1.** Express confidence in their capability to learn novel theory and practice recommendations. |  | **O2.E1.** Have access to tailored educational materials  **O2.E2.** Have time available to attend educational meetings or read/listen/watch educational materials.  **O2.E3.** re involved in discharge communication by the hospital |
|  |  |  |  |  |  |  |
| **Microbiologist (adopter)** |  |  |  |  |  |  |
|  |  |  |  |  |  |  |
| **O1. Agree to change the local antibiotic guidance protocol, incorporating neonatal oral switch therapy.** | **O1.K1.** Describe the key evidence supporting oral switch therapy  **O1.K2.** Describe the type, dose and formula of antibiotics needed for oral switch therapy | **O1.R1.** Acknowledge that pediatricians recognize culture-negative sepsis and treat such cases with antibiotics | **O1.A1.** Belief that oral switch therapy is a safe and effective alternative for intravenous therapy  **O1.A2.** Expect that oral switch therapy will result in shorter hospital stay and increased patient comfort  **O1.A3.** Expect that oral switch therapy will not result in increased antibiotic prescriptions |  | **O1.H1.** Refrain the current standard practice of advising intravenous therapy for all cases of culture-negative sepsis. |  |

**Table S4.** Implementation strategies

| **Target group of strategy** | ***Determinants*** | ***Method*** | **Implementation strategy** | **Executer** | **Duration/frequency** |
| --- | --- | --- | --- | --- | --- |
| **Paediatricians, paediatric residents, physician assistants, nurses, primary care maternity nurses, midwives, pharmacists, microbiologists** | Knowledge,  Awareness and Risk Perception,  Attitude, beliefs, outcome expectations**,**  Skills, capability, self-efficacy  Habitual behaviour,  Environmental factors | *Identify and prepare champions**  *Advocacy and lobbying*  *Remind clinicians**  *Modeling*  *Provide ongoing consultation** | **Appointing local implementation champions**  Two paediatricians and one neonatal nurse, who are seen as ‘opinion leaders’ by their colleagues, are appointed as local champions.  Task list:  Pre-implementation tasks   1. Briefing and discussing the interventions with the paediatric specialist group and microbiologist 2. Reviewing resource availability with the clinical chemistry laboratory (PCT determination equipment) and pharmacy (amoxicillin suspension) 3. Informing and engaging the obstetric department and obstetric partnerships (VSV) 4. Adapting local medical and nursing guidelines to incorporate the centrally prescribed interventions. 5. Tasks related to specific implementation strategy components   Ongoing tasks   - Repeatedly remind the team to adhere to the guideline with the novel interventions - Share knowledge on the topic with their colleagues - Identify novel barriers of implementation and share this with the central team to create a solution. - Serving as the primary point of contact for all queries from various stakeholders. | Appointment: collaboratively by paediatric specialist team and nursing team.  Briefing: by central research team  Executing tasks of task list: local implementation champions | Appointment and briefing: during implementation preparation period  Pre-implementation tasks: during implementation preparation period  Ongoing tasks: during the entire roll out of project |
| **Paediatricians, paediatric residents** | *Knowledge,*  *Awareness and Risk Perception,*  *Attitude, beliefs, outcome expectations,*  *Skills, capability, self-efficacy,*  *Habitual behaviour* | *Persuasive communication*  *Consciousness raising*  *Advance organizers*  *Discussion*  *Arguments*  *Advance organizers* | **Paediatric education-discussion kick-off session and ready-to-use presentation template**  Education  *Presentation of current numbers and room for improvement*  Persuasive messages of numbers on current Dutch treatment rates, EOS incidence, risk of antibiotic therapy and variation in clinical practice during the educational discussion session.  *Presentation of novel interventions (educational)*  Schematic overview of novel interventions while using current guideline as steppingstone for novel knowledge with links to relevant articles/knowledge sources per topic.  Discussion  *Discussion session*  Discussion will be started based on discussion point brought up by the participants, or will be initiated by discussing the common pitfalls of the interventions by the implementation leaders.  *Presentation of arguments based on scientific evidence*  Evidence on the safety, effectiveness and relative advantages of the three clinical interventions will be shared in response to attitudes, beliefs and expectations of the respective group, thus tailored to the individual needs.  *Sharing presentation template with links to knowledge sources*  Presentation template with schematic overview of the interventions and relevant knowledge sources is shared with all paediatricians and paediatric residents. | Local paediatric champions together with central implementation leader*.    **Local implementation team will provide presentation template and give presentation. The discussion will be led by local paediatric champions in presence of central implementation leader. Local paediatric champion will repeat educational session if needed.* | At least one session at start of active implementation period  Ready-to-use presentation template will made available for all pediatricians and paediatric residents during entire rollout of the project |
| **Neonatal nurses** | *Knowledge,*  *Awareness and Risk Perception,*  *Skills, capability, self-efficacy*  *Attitude, beliefs, outcome expectations,* | *Train-the-trainer*  *Guided practice*  *Advance organizers* | **Nurse peer-to-peer education and ready-to-use presentation template**  -Nurse education through peer-to-peer learning.  -Local implementation team appoints nurse that will be repsonsible for education  -Responsible nurse receives extensive briefing on oral switch therapy and tasks.  -Education methods: bedside teaching, online/live sessions, or instructional email (dependent on local education routines) by educational nurse and peers  -Ready-to-use presentation template is shared with all nurses | Appointment of education nurse: local implementation team  Providing ready-to-use presentation template: central research team  Providing education to nursing team: educational nurse | Appointment of education nurse(s): during implementation preparation period  All nurses receive education within the first two months after implementation started.  Ready-to-use presentation template will be made available for all nurses and will be available during the entire rollout of the project. |
| Maternity nurses | *Knowledge,*  *Skills, capability, self-efficacy,*  *Attitude, beliefs, outcome expectations* | *Develop and distribute educational materials** | **Maternity nurse knowledge document and newsletter**  -Informative document on oral switch therapy available on Knowlegde Centre for Maternity Care’s (KCKZ) website, covering early-onset sepsis, oral switch therapy, care responsibilities and safety precautions.  - Information and link to informative document distributed to maternity nurses via KCZK email newsletter. | Providing the knowledge document: central research team  Uploading on website and sharing in newsletter: policy officer of KCZK | Document uploaded and disseminated: during implementation preparation period |
| Parents | *Knowledge,*  *Awareness and Risk Perception,*  *Attitude, beliefs, outcome expectations,*  *Skills, capability, self-efficacy* | *Guided practice*  *Develop and distribute educational materials** | **Parent guided-practice, instruction folder and medication scheme checklist**  -Parents receive training from a neonatal nurse on administering oral antibiotics.  -Parents practice administering at least one dose under nurse guidance in the hospital.  - Parents receive instructional folder with detailed guidance, including missed dose instructions, warning signs, contact information and a medication schedule checklist for dose administration at home.  -Folder and checklist available in Dutch and English, B2 language level | Providing template for parent instructional folder: central research team  Adapting and printing local parent instruction folder: local implementation team  Providing parents with training and folder: neonatal nurses | Providing template for parent instructional folder, adapting and printing: during implementation preparation period  Providing parents with training and folder: at every discharge of neonate on IV-to-oral switch therapy |
| Primary: parents  Secondary: maternity nurses, neonatal nurses | *Knowledge,*  *Awareness and Risk Perception,*  *Attitude, beliefs, outcome expectations,*  *Skills, capability, self-efficacy* | *Using imagery*  *Develop and distribute educational materials** | **Oral switch therapy instructional video**  -Instructional video on oral switch therapy, administration, safety, and when to contact the pediatric department is shared with parents at discharge  -Video accessible via a free streaming platform and project website, linked through a QR code in the parent instructional folder.  -Available in Dutch and English, B2 language level | Developing and uploading video on streaming platform: central research team  Sharing video with parents at discharge: neonatal nurses | Developing and uploading video on streaming platform: during implementation preparation period  Sharing video with parents at discharge: at every discharge on IV-to-oral switch therapy |
| Primary: paediatricians, paediatric residents  Secondary: neonatal nurses, gynaecologists, microbiologists | *Knowledge,*  *Awareness and Risk Perception,*  *Attitude, beliefs, outcome expectations****,***  *Habitual behaviour,* | *Role models*  *Develop and distribute educational materials** | **Evidence based podcast series on early onset sepsis with role models**  -Recording 5 podcast episodes with role models and experts in the field of EOS  -Publishing podcast on free streaming platform  -Sharing link to episodes with the personal email addresses of all paediatricians and paediatric residents of participating sites  -Sharing information and link to podcast in an article on the website of the National Association of Paediatrics (NVK, [www.nvk.nl](http://www.nvk.nl)) and the NVK newsletter sent to all paediatricians | Recording and publishing: central research team  Sharing: local implementation team and communication department of NVK | Recording: during the implementation preparation period  Publishing and sharing: during the active implementation period |
| Paediatricians | *Environmental factors*  *Skills, capability, self-efficacy* | *Technical assistance/facilitation* | **Facilitation of HIX calculator integration in EPD**  -Coordinating EOS calculator availability in Hix at every site that uses the HiX EHR  -Sharing instructions on how to activate and use the EOS calculator in daily practice  -Technical assistance in case issues emerge | Central research team in collaboration with local implementation team and ICT department | Availability coordination and sharing instructions during implementation period  Technical assistance during entire rollout of the project |

*Implementation strategies are based on Taxonomy of Behavioural Change and CFIR-ERIC matching tool[3,4] ERIC strategies are indicated with a (*).*

**References**

[1] Veen LEJ van, Janssen SWCM, Tramper-Stranders GA, Achten NB, Rossum AMC van, Plotz FB, et al. Facilitators and barriers when implementing antibiotic stewardship interventions in neonates at risk of early-onset sepsis. MedRxiv 2025:2025.03.31.25324838. https://doi.org/10.1101/2025.03.31.25324838.

[2] Damschroder LJ, Reardon CM, Widerquist MAO, Lowery J. The updated Consolidated Framework for Implementation Research based on user feedback. Implementation Science 2022;17. https://doi.org/10.1186/s13012-022-01245-0.

[3] Kok G, Gottlieb NH, Peters GJY, Mullen PD, Parcel GS, Ruiter RAC, et al. A taxonomy of behaviour change methods: an Intervention Mapping approach. Health Psychol Rev 2015;10:297. https://doi.org/10.1080/17437199.2015.1077155.

[4] Powell BJ, Waltz TJ, Chinman MJ, Damschroder LJ, Smith JL, Matthieu MM, et al. A refined compilation of implementation strategies: Results from the Expert Recommendations for Implementing Change (ERIC) project. Implementation Science 2015;10. https://doi.org/10.1186/s13012-015-0209-1.
