# Supplementary material for: Implementation strategy for an antibiotic stewardship bundle to promote optimal treatment choices in neonates with suspected early-onset sepsis (Protect-Neo): a study protocol for a multicentre, prospective interrupted time series and before-after study
Source: BMJ Open. 2025 Nov 4;15(11):e103368. doi: 10.1136/bmjopen-2025-103368 (PMC12588035; doi:10.1136/bmjopen-2025-103368)
Supplement: online supplemental file 3 [file bmjopen-15-11-s003.docx]

**Supplemental file 3 – Study outcomes**

| **Table S5.** Study outcomes | | | | |  |  |
| --- | --- | --- | --- | --- | --- | --- |
| **Outcome** | **Definition** | **Study definition** | **Data source** | **Research population** | **Level of analysis** | **Reporting measure** |
| **Primary outcome** | | | | | | |
| Antibiotic exposure in days of therapy (DOT) | CDC: The aggregate sum of days for which any amount of a specific antimicrobial agent was administered to individual patient [1].  AENEAS: The sum of all antibiotic days (calendar days with at least one dose) for all neonates that were started on therapy in the first week of life [2]. | The sum of all antibiotic days (calendar day with at least one dose of antibiotics) for all neonates that were started on therapy in the first 72 hours after birth. | QUANTITATIVE  Electronic health record data | Live-born neonates born with a gestational age of 34 weeks or more | Per site and all sites combined | QUANTITATIVE  Antibiotic days/1000 live births (95% CI) on any antibiotic and per specific antimicrobial agent |
| **Secondary implementation outcomes** | | | | | | |
| **Adoption of the antibiotic stewardship interventions in local policy** | The intention, initial decision or action to employ an intervention or evidence-based practice. [3] | Integration of the antibiotic stewardship intervention recommendations in the formal local protocol | QUANTITATIVE  Departments’ protocols  Logbook  QUALITATIVE  Focus group evaluation sessions | Neonatology department | All sites combined | QUANTITATIVE  Number of departments that integrated the interventions in their formal local protocol  QUALITATIVE  Departments’ reported reasons for non-adoption |
| **Fidelity to the antibiotic stewardship interventions** | The degree to which an intervention or strategy was implemented as it was prescribed in the original protocol or as it was intended by the program developers [3]. | **EOS calculator**  EOS calculator use in neonates with risk factors of early-onset sepsis | QUANTITATIVE  Electronic health record data | Neonates born with a gestational age of 34 weeks or more | Per site and all sites combined | QUANTITATIVE  Proportion (%) of eligible neonates |
|  |  | **PCT-guided therapy**  Application of PCT guided therapy in low or medium risk neonates |  |  |  |  |
|  |  | **Oral switch therapy**  Prescription of oral antibiotics in neonates that are treated with a full course of antibiotics, that have negative blood culture, good clinical status and increased infection markers. |  |  |  |  |
| **Acceptability of the antibiotic stewardship interventions** | The extent to which stakeholders perceive that the clinical intervention or strategy is agreeable, palatable, or satisfactory [3]. | The extent to which stakeholders perceive the antibiotic stewardship interventions as an acceptable and a satisfactory choice of policy | QUANTITATIVE  Survey  QUALITATIVE  Field notes  Individual interviews  Focus group evaluation sessions | E, P, O: Paediatricians, paediatric residents, neonatology nurses | All sites combined | QUANTITATIVE  Total score on AIM questionnaire[4]  QUALITATIVE  Reported acceptability, regarding affective attitude, burden, ethicality, intervention coherence, opportunity costs, perceived effectiveness, self-efficacy [5]. |
|  |  |  |  | O: Maternity nurses, parents |  |  |
| **Feasibility of the antibiotic stewardship interventions** | The extent to which a new treatment, or an innovation, can be successfully used or carried out within a given agency or setting [3]. | The extent to which stakeholders think the interventions can be successfully used in their setting, including reasons for not being able to adhere to the clinical protocol | QUANTITATIVE  Survey  QUALITATIVE  Field notes  Individual interviews  Focus group evaluation sessions | E, P, O: Paediatricians, paediatric residents, neonatology nurses | All sites combined | QUANTITATIVE  total score on FIM questionnaire[4]  QUALITATIVE  Reported feasibility (costs, available resources, technical equipment) |
|  |  |  |  | O: Maternity nurses, parents |  |  |
| **Fidelity to the implementation strategies** | The degree to which an intervention or strategy was implemented as it was prescribed in the original protocol or as it was intended by the program developers [3]. | The extent to which the individual components of the implementation strategy were executed as prescribed by the central research team. | QUANTITATIVE  Task checklist (part of logbook)  Survey  QUALITATIVE  Field notes  Focus group evaluation sessions  Individual interviews | Paediatricians and paediatric residents (C 1-3, 8), neonatology nurses (C1,4,6,7), maternity nurses (C1,5,6,7), parents (C6,7) | Per site and all sites combined | QUANTITATIVE  Number of completed steps per strategy component  Attendance/education registration list  QUALITATIVE  Degree to which the local implementation leaders and education nurse executed their tasks properly as reported by other team members/ colleagues |
| **Appropriateness of the**  **implementation strategies** | Appropriateness is the perceived fit, relevance, or compatibility of the innovation or evidence-based practice for a given practice setting, provider, or consumer[3]. | The extent to which stakeholders perceive the individual components of the implementation strategies as relevant for their setting | QUANTITATIVE  Survey  QUALITATIVE  Field notes  Focus group evaluation sessions  Individual interviews | Paediatricians and paediatric residents (C 1-3, 8), neonatology nurses (C1,4,6,7), maternity nurses (C1,5,6,7), parents (C6,7) | All sites combined | QUANTITATIVE  5 point Likert-scale on relevance  QUALITATIVE  Reported opinions of stakeholders about helpfulness and relevance of each implementation strategy component |
| **Secondary effectivity outcomes (clinical)** | | | | | | |
| **Proportion of neonates receiving antibiotics** |  | Neonate started on antibiotics in the first 72 hours of life | QUANTITATIVE  Electronic health record data | Live-born neonates born with a gestational age of 34 weeks or more | Per site and all sites combined | Proportion (%) |
| **Proportion of neonates receiving antibiotics per therapy duration category** |  | Neonate started on antibiotics in the first 72 hours of life, receiving antibiotics for ≤36 hours, 36-48 hours, 48-72 hours, ≥5 days or ≥7 days. |  | Neonates that received antibiotic therapy for suspected EOS |  | Proportion (%) |
| **Duration of therapy** |  | The time period in days that a neonate receives at least 1 dose of antibiotics |  | Neonates that received antibiotic therapy for suspected EOS |  | Duration in days |
| **Proportion neonates receiving IV-to-oral switch therapy** |  | Neonates switching to oral antibiotics within 72 hours after blood culture collection |  | Neonates with a negative blood culture that received a full course of antibiotics (at least 5 days) |  | Proportion (%) |
| **Cumulative length of hospital stays** |  | The total time period that neonates are hospitalized, starting from the day they are first admitted until 28 days after birth |  | Neonates that received antibiotic therapy for suspected EOS |  | Duration in days |
| **Cumulative number of laboratory tests** |  | The number of blood tests that were performed, including C-reactive protein (CRP), procalcitonin (PCT) and white blood cell count (WBC) |  | Neonates that received antibiotic therapy for suspected EOS |  | Proportion (%) |
| **Cumulative bacterial (re)infection rate** |  | All infections or reinfections, defined as elevated C-reactive protein concentrations ≥10 mg/L or elevated procalcitonin concentrations ≥0·5 ng/mL) with a need for prolonged antimicrobial therapy (>48h), within 28 days after birth |  | Neonates that were either observed or received antibiotic therapy for suspected EOS |  | Proportion (%) |
| **Cumulative readmission rate** |  | Readmission to the hospital within 28 days after birth |  | Neonates that were either observed or received antibiotic therapy for suspected EOS |  | Proportion (%) |
| **EOS incidence** |  | Neonates with bacterial growth in the blood culture, identified by the clinician as pathogen causing EOS |  | Live-born neonates born with a gestational age of 34 weeks or more |  | Proportion (%) |
| **Pathogen determination and susceptibility pattern** |  | Pathogen determination and susceptibility pattern of all positive cultures drawn within the first 28 days after birth |  | Live-born neonates born with a gestational age of 34 weeks or more |  | Descriptive |
| **Infection related mortality** |  | Neonate who died within 28 days of birth due to an infection-related cause. |  | All neonates born at a gestational age of 34 weeks or more |  | Proportion (%) |
| **Presence of practice and knowledge-gaps** |  | Knowledge of and practices in early-onset sepsis care (including EOS incidence, antibiotic use, biomarkers and blood cultures) | QUANTITATIVE  Survey | Paediatricians, paediatric residents | All sites combined | Proportion (%) per topic |

**Referentces**

[1] Center for Disease Control and Prevention. Antimicrobial Use and Resistance (AUR) Module 2025.

[2] Giannoni E, Dimopoulou V, Klingenberg C, Navér L, Nordberg V, Berardi A, et al. Analysis of Antibiotic Exposure and Early-Onset Neonatal Sepsis in Europe, North America, and Australia. JAMA Netw Open 2022;5:E2243691. https://doi.org/10.1001/jamanetworkopen.2022.43691.

[3] Proctor E, Silmere H, Raghavan R, Hovmand P, Aarons G, Bunger A, et al. Outcomes for Implementation Research: Conceptual Distinctions, Measurement Challenges, and Research Agenda. Administration and Policy in Mental Health and Mental Health Services Research 2011;38:65–76. https://doi.org/10.1007/s10488-010-0319-7.

[4] Weiner BJ, Lewis CC, Stanick C, Powell BJ, Dorsey CN, Clary AS, et al. Psychometric assessment of three newly developed implementation outcome measures. Implementation Science 2017;12:1–12. https://doi.org/10.1186/S13012-017-0635-3/TABLES/3.

[5] Sekhon M, Cartwright M, Francis JJ. Acceptability of healthcare interventions: An overview of reviews and development of a theoretical framework. BMC Health Serv Res 2017;17:1–13. https://doi.org/10.1186/S12913-017-2031-8/TABLES/3.
