# Supplementary material for: Implementation strategy for an antibiotic stewardship bundle to promote optimal treatment choices in neonates with suspected early-onset sepsis (Protect-Neo): a study protocol for a multicentre, prospective interrupted time series and before-after study
Source: BMJ Open. 2025 Nov 4;15(11):e103368. doi: 10.1136/bmjopen-2025-103368 (PMC12588035; doi:10.1136/bmjopen-2025-103368)
Supplement: online supplemental file 4 [file bmjopen-15-11-s004.docx]

**Survey post-implementation – Paediatricians – Protect-NEO**

***Survey in Castor EDC, translated from Dutch***

**General and Incidence**

- 1. **What is the incidence of proven EOS among neonates born in the Netherlands?**

☐ ~1 in 10,000

☐ ~1 in 1,000

☐ ~1 in 100

☐ ~1 in 10

☐ I do not know

- 1. ***How many neonates, born in a Dutch hospital, receive antibiotics within the first 72 hours after birth?***

☐ ~3–4 in 10,000

☐ ~3–4 in 1,000

☐ ~3–4 in 100

☐ ~3–4 in 10

☐ I do not know

**EOS Calculator**

**2.1 Is the neonatal early-onset sepsis (EOS) calculator formally included in your department’s local policy for neonates at risk of EOS?**

☐ Yes

☐ No

☐ I do not know

**2.2.1 How often do you use the EOS calculator when deciding whether to start antibiotics in a neonate with increased risk of infection?**

**2.2.2. How often do you follow the advice of the EOS calculator once you have completed it?**

☐ Never

☐ Rarely

☐ Sometimes

☐ Often

☐ Always

☐ Not applicable

**2.3 Indicate the extent to which you agree with the following statements**

***2.3.1 The EOS calculator has my approval***

***2.3 I find the EOS calculator appealing***

***2.4 I like the EOS calculator***

***2.5 I welcome the use of the EOS calculator in practice***

☐ Strongly disagree

☐ Disagree

☐ Neither agree nor disagree

☐ Agree

☐ Strongly agree

**2.4 Indicate the extent to which you agree with the following statements**

**2.4.1. Using the EOS calculator seems implementable.**

**2.4.2. Using the EOS calculator seems possible**

**2.4.3. Using the EOS calculator seems doable.**

**2.4.4. The EOS calculator seems easy to use.**

☐ Strongly disagree

☐ Disagree

☐ Neither agree nor disagree

☐ Agree

☐ Strongly agree

**3. Policy on Starting Antibiotics**

**3.1 Which additional diagnostics do you usually perform at the start of antibiotics (t=0)?**

☐ None

☐ CRP

☐ Procalcitonin (PCT)

☐ White blood cell count

☐ Other: …

*3.1.2 Do you wait for the CRP result before deciding to start antibiotics?*

☐ Yes

☐ No

☐ Sometimes

*3.1.3 Do you wait for the PCT result before deciding to start antibiotics?*

☐ Yes

☐ No

☐ Sometimes

*3.1.4 Do you wait for the white blood cell count before deciding to start antibiotics?*

☐ Yes

☐ No

☐ Sometimes

**4Blood Culture**

**4.1 What is the minimum blood volume required for a reliable neonatal blood culture?** ………… mL

**4.2 If a blood culture bottle cannot immediately be placed in the incubator (e.g. out-of-hours), where should it be stored?**

☐ At room temperature

☐ In a warming cabinet (± 38 °C)

☐ Both are acceptable

☐ I do not know

**4.3 After how many hours can you be reasonably certain (±95%) that a properly taken blood culture will remain negative?**

………… hours

**4.4 After how many hours do you usually decide in practice whether to stop antibiotics when a blood culture remains negative?**

………… hours

**5. Biomarkers**

**5.1.1 CRP can be used effectively for:**

**5.1.2 Procalcitonin can be used effectively for:**

**5.1.3 White blood cell count can be used effectively for:**

☐ Excluding infection

☐ Detecting infection

☐ Both

☐ Neither

☐ I do not know

**5.4 At which time points after starting antibiotics do you usually measure CRP?**

☐ Not measured

☐ 6 hours

☐ 12 hours

☐ 24 hours

☐ 36 hours

☐ 48 hours

☐ Other: …

**5.5 From which CRP value would you continue treatment, regardless of negative blood culture and good clinical condition?**

………… mg/L

**6. PCT-guided therapy**

**6.1 Is procalcitonin-guided stopping of antibiotics (NeoPIns study) included in your local EOS policy?**

☐ Yes

☐ No

☐ I do not know

**6.2.1 How often do you measure PCT at t=12 and t=24 hours in neonates with low/moderate risk?**

**6.2.2 How often do you stop antibiotics directly (without awaiting blood culture) when PCT is low twice?**

☐ Never

☐ Rarely

☐ Sometimes

☐ Often

☐ Always

☐ Not applicable

**6.3 Indicate the extent to which you agree with the following statements**

**6.3.1 PCT-guided therapy has my approval**

**6.3 I find PCT-guided therapy appealing**

**6.4 I like PCT-guided therapy**

**6.5 I welcome the use of PCT-guided therapy in practice**

☐ Strongly disagree

☐ Disagree

☐ Neither agree nor disagree

☐ Agree

☐ Strongly agree

**6.4 Indicate the extent to which you agree with the following statements**

**6.4.1. PCT-guided therapy seems implementable.**

**6.4.2. PCT-guided therapy seems possible**

**6.4.3. PCT-guided therapy seems doable.**

**6.4.4. PCT-guided therapy seems easy to use.**

☐ Strongly disagree

☐ Disagree

☐ Neither agree nor disagree

☐ Agree

☐ Strongly agree

**7. Oral Switch Therapy**

**7.1 Is oral switch therapy (RAIN study) included in your local neonatal sepsis policy?**

☐ Yes

☐ No

☐ I do not know

**7.2 How often do you prescribe oral antibiotics after 36–48 hours IV treatment in eligible neonates?**

☐ Never

☐ Rarely

☐ Sometimes

☐ Often

☐ Always

☐ Not applicable

**7.3 Indicate the extent to which you agree with the following statements**

**7.3.1 IV-oral switch therapy has my approval**

**7.3 I find IV-oral switch therapy appealing**

**7.4 I like IV-oral switch therapy**

**7.5 I welcome the use of IV-oral switch therapy in practice**

☐ Strongly disagree

☐ Disagree

☐ Neither agree nor disagree

☐ Agree

☐ Strongly agree

**7.4 Indicate the extent to which you agree with the following statements**

**7.4.1. IV-oral switch therapy seems implementable.**

**7.4.2. IV-oral switch therapy seems possible**

**7.4.3. IV-oral switch therapy seems doable.**

**7.4.4. IV-oral switch therapy seems easy to use.**

☐ Strongly disagree

☐ Disagree

☐ Neither agree nor disagree

☐ Agree

☐ Strongly agree

**8. Implementation strategies**

**8.1.Please indicate how relevant the following implementation strategies have been in recent years for implementing the bundle over EOS interventions:**

- **Local implementation champions who were responsible for carrying out the implementation and served as a point of contact**
- **Education-discussion session with ready-to use educational materials**
- **Knowledge podcast**
- **Integrating the interventions (EOS calculator) into the electronic health record**

☐ Not at all relevant
☐ Slightly relevant
☐ Moderately relevant
☐ Very relevant
☐ Extremely relevant

**8. Work Experience**

**8.1 Please indicate which applies to you**

☐ General paediatrician

☐ Subspecialist paediatrician

☐ Paediatric trainee (resident)

☐ Paediatric resident not in training

☐ Neonatology nurse practitioner

☐ Other: …

**8.2 How many years have you been working in your current role (any workplace)?**

☐ 0–5 years

☐ 6–10 years

☐ 10–15 years

☐ >15 years

**8.3 What is your age?**

………… years
