# Supplementary material for: Implementation strategy for an antibiotic stewardship bundle to promote optimal treatment choices in neonates with suspected early-onset sepsis (Protect-Neo): a study protocol for a multicentre, prospective interrupted time series and before-after study
Source: BMJ Open. 2025 Nov 4;15(11):e103368. doi: 10.1136/bmjopen-2025-103368 (PMC12588035; doi:10.1136/bmjopen-2025-103368)
Supplement: online supplemental file 6 [file bmjopen-15-11-s006.docx]

**Survey post-implementation – Nurses – Protect-NEO**

**1. Oral Switch Therapy**

**1.1 Is oral switch therapy (RAIN study) included in your local neonatal sepsis policy?**

☐ Yes

☐ No

☐ I do not know

**1.2 In case a neonate is going home with oral antibiotics, do you or your colleague give an instruction on how to administer antibiotics?**

☐ Never

☐ Rarely

☐ Sometimes

☐ Often

☐ Always

☐ Not applicable

**1.2 In case a neonate is going home with oral antibiotics, do you or your colleague make sure the parents practiced administering antibiotics to their neonate at the ward?**

☐ Never

☐ Rarely

☐ Sometimes

☐ Often

☐ Always

☐ Not applicable

**1.3 Indicate the extent to which you agree with the following statements**

**1.3.1 IV-oral switch therapy has my approval**

**1.3 I find IV-oral switch therapy appealing**

**1.4 I like IV-oral switch therapy**

**1.5 I welcome the use of IV-oral switch therapy in practice**

☐ Strongly disagree

☐ Disagree

☐ Neither agree nor disagree

☐ Agree

☐ Strongly agree

**1.4 Indicate the extent to which you agree with the following statements**

**1.4.1. IV-oral switch therapy seems implementable.**

**1.4.2. IV-oral switch therapy seems possible**

**1.4.3. IV-oral switch therapy seems doable.**

**1.4.4. IV-oral switch therapy seems easy to use.**

☐ Strongly disagree

☐ Disagree

☐ Neither agree nor disagree

☐ Agree

☐ Strongly agree

- 1. **Please indicate how relevant the following implementation strategies have been in recent years for implementing IV-to-oral switch therapy in neonates:**
- **Local implementation champions who were responsible for carrying out the implementation and served as a point of contact**
- **Ready-to use educational materials for nurses**
- **Guided practice for parents, along with an instruction leaflet and checklist**
- **Parent instruction video**

☐ Not at all relevant
☐ Slightly relevant
☐ Moderately relevant
☐ Very relevant
☐ Extremely relevant
