# Supplementary material for: Implementation strategy for an antibiotic stewardship bundle to promote optimal treatment choices in neonates with suspected early-onset sepsis (Protect-Neo): a study protocol for a multicentre, prospective interrupted time series and before-after study
Source: BMJ Open. 2025 Nov 4;15(11):e103368. doi: 10.1136/bmjopen-2025-103368 (PMC12588035; doi:10.1136/bmjopen-2025-103368)
Supplement: online supplemental file 7 [file bmjopen-15-11-s007.docx]

**Interview Guide Protect-NEO – Implementation Evaluation**

**Introduction** (what should be mentioned)

- Welcome: today we will jointly review the implementation of the new EOS protocol, including the EOS calculator, PCT-guided discontinuation of antibiotics, and IV-to-oral switch therapy.
- Structure:
  - Discuss each intervention separately: inclusion in protocol/daily practice, acceptability, feasibility, and support.
  - Afterwards, review the implementation process (insofar as not yet discussed): which strategies have been effective, and which have not?
- Confidentiality: This session will be recorded; all data will be processed anonymously.
- One slide summarising the three interventions as a refresher.
- Explanation of using MENTIMETER: to start the discussion. No perfect answers required.

**Opening Question**

[MENTIMETER] Indicate which answer applies since the introduction of the revised EOS protocol:

- I prescribe more antibiotics
- I prescribe fewer antibiotics
- I prescribe the same amount of antibiotics
- Can you explain why you think this?

**Intervention-Specific Evaluation**

- How are you currently using the EOS calculator in clinical practice?
  - For which group of neonates? Duration of observation? Guidance of policy?
- [MENTIMETER] Would you describe the use of the EOS calculator as your standard policy?
  - How much support is there within the team?
  - What is the main reason it has or has not been (partly) successful? What has contributed to its success? (Are there any strategies to mention?)
- Is it feasible to use the calculator in practice?
  - [MENTIMETER] What are the main barriers to using the EOS calculator?
    - Observation capacity, data availability, etc.
- Would you like to see this tool included in the national guideline?
  - If so, under which conditions?

**PCT-Guided therapy**

- How do you currently use PCT in clinical practice?
  - After how many hours is it drawn? Discontinuation before the blood culture? What if results are high?
- [MENTIMETER] Would you describe the use of PCT-guided therapy as your standard policy?
  - How much support is there within the team?
  - What is the main reason it has or has not been (partly) successful? What has contributed to its success? (Are there any strategies to mention?)
- Is it always possible to determine PCT/ is it feasible in your department?
- [MENTIMETER] What are the biggest obstacles in using PCT?
  - - Costs, time for determination, availability of material, blood collection rounds
- Would you like recommendations regarding the use of PCT included in the national guideline?
  - If so, under which conditions?

**Oral Switch Therapy**

- How do you currently apply oral switch therapy in practice (adoption)?
  - After how many hours? For which patients? What dosage and type of antibiotics?
  - Explanation to parents? Handover to primary care?
- [MENTIMETER] Would you describe prescribing oral switch therapy as your standard policy?
  - How much support is there within the team?
  - What is the main reason it has or has not been (partly) successful? What has contributed to its success? (Are there any strategies to mention?)
- Is prescribing oral switch therapy always feasible in your department?
- [MENTIMETER] What are the primary barriers to applying IV-to-oral switch therapy?
- How is handover to primary care ensured?
  - Suspension availability, communication with primary care, etc.
- Would you like oral switch therapy included in the national guideline?
  - If so, under which conditions?

**General evaluation**

- [MENTIMETER]: If another hospital wants to implement this protocol in their department, what actions are needed for successful implementation?
- [MENTIMETER]: How useful did you find the following implementation strategies in supporting the rollout of the new protocol? Rate from 1-10:
- Educational and discussion sessions by the lead investigator (kick-off)
- Podcast about early-onset sepsis (or: not listened)
- Local champions
- Integration of the calculator in HiX (or not applicable)
- Parent instructions (leaflet, nurse explanation)
- Do you have additional suggestions for strategies?
- Are you as a department continuing with this protocol?
  - If so, why?
  - If not, why not?

**Other questions about the implementation process** (depending on what has already been discussed):

- What was less successful? Why did implementation not take off immediately?
- What would you approach differently at the next implementation?
- How did you reach colleagues who missed training?
- How useful was the educational session for doctors/nurses?
- Were local champions visible enough? Were they adequately supported?
- Did the podcast contribute to knowledge or support?
- To what extent does the EHR sufficiently support the new policy?

**Closing**

- What have we not discussed, but should have been?
