# Supplementary material for: Implementation strategy for an antibiotic stewardship bundle to promote optimal treatment choices in neonates with suspected early-onset sepsis (Protect-Neo): a study protocol for a multicentre, prospective interrupted time series and before-after study
Source: BMJ Open. 2025 Nov 4;15(11):e103368. doi: 10.1136/bmjopen-2025-103368 (PMC12588035; doi:10.1136/bmjopen-2025-103368)
Supplement: online supplemental file 8 [file bmjopen-15-11-s008.docx]

# Interview Guide – Parents – Protect-NEO

## Introduction

- Introduce yourself and explain that you are calling for an interview as part of the Protect-Neo study.
- Confirm the child’s name and date of birth.
- Aks consent for the conversation being recorded after explaining that all information will be treated confidentially.

## General

- How are [Child’s name] and you doing now?
- How long have you been home?
- For how many days did [Child’s name] receive antibiotics at home?

## Experiences with oral antibiotics at home

- How did you feel at first when you heard [Child’s name] could go home with oral antibiotics?
- Did you receive an explanation or education in the hospital about giving antibiotics to your newborn?
  - Did you get the opportunity to practice administering antibiotics with a nurse before going home?
- How did you feel at first when you heard you could go home with oral antibiotics?
- How did administering the antibiotic drink at home go?
- Did [Child’s name] have difficulties with spitting up or swallowing?
- Were you able to give all the doses?
- Were there any questions at home that you would have preferred to ask while still in the hospital?
- Looking back, would you have preferred going on with intravenous antibiotics in the hospital or oral antibiotics at home? Why?

## Information materials: leaflet and video

- Did you receive a leaflet with information on early onset sepsis?
  - If yes, was the information leaflet clear?
    - Readability, missing information
- Did you find the leaflet useful?
  - Why or why not?
  - Would you recommend giving the leaflet to parents in the future?
- Did you use the checklist that accompanied the leaflet?
  - Did you consider the checklist to be useful/necessary?
  - Would you recommend giving the checklist to parents in the future?
- Did you receive a link to the instructional video? Did you watch it?
  - If yes: was the video clear?
    - Understandability, missing information
- Did you find the video useful?
  - Why or why not?
  - Would you recommend other parents to watch the video?
- What, in your opinion, is the best way to receive information about administering therapy at home?

## Contact with healthcare providers

- Did any questions come up at home that you would have liked to ask in the hospital?
- Was it clear to you when and how you should contact the pediatrician?
- How did you experience the support of maternity care?
  - Was the maternity nurse familiar with administering antibiotics at home?

## Suggestions for the future

- What advice would you give to hospital staff to improve the care of babies receiving antibiotics for infections?
- What advice would you give to other parents whose baby first receives antibiotics through an IV in the hospital and later orally at home?
