# Supplementary material for: Implementation strategy for an antibiotic stewardship bundle to promote optimal treatment choices in neonates with suspected early-onset sepsis (Protect-Neo): a study protocol for a multicentre, prospective interrupted time series and before-after study
Source: BMJ Open. 2025 Nov 4;15(11):e103368. doi: 10.1136/bmjopen-2025-103368 (PMC12588035; doi:10.1136/bmjopen-2025-103368)
Supplement: online supplemental file 9 [file bmjopen-15-11-s009.docx]

Interview Guide – Maternity Nurses – Protect-NEO

**Introduction**

- Introduce yourself and explain that you are conducting an interview as part of the Protect-NEO research study.
- Ask consent for the conversation being recorded after explaining that all information will be treated confidentially.

**General**

- How did you experience providing care to a family whose baby was discharged with oral antibiotics?
- How did you see your role in supporting the parents in giving oral antibiotics?
- Did you feel confident in guiding or assisting the parents with antibiotic administration? Why/why not?
- Were there specific tasks or responsibilities you took on (e.g., observing, reminding, checking doses)?
- Did caring for a neonate on antibiotics increase your workload or that of the midwife?

**Information and preparation**

- Were you informed by the hospital that the neonate was discharged with antibiotics?
  - If yes, was the information you received from the hospital clear and sufficient?
- Did you feel you had enough knowledge to support the parents in giving antibiotics?
- Are you aware that there is a protocol about neonates going home with antibiotics on the KCKZ website?
  - Do you find this protocol clear and practical?
    - If not, what information or training would have helped you?
- Did the parents ask you questions about antibiotics at home that you were not able to answer?

**Information materials: leaflet and video**

- Were you aware that parents received a leaflet and/or video about oral antibiotics?
- Did you have the opportunity to look at these materials yourself?
  - If yes: did you find them clear and useful?
  - If not: would you like to have access to these materials as well, in order to better support parents?

**Contact with the paediatrics department**

- Was it clear how and when parents should contact the paediatrician, and did you feel able to support them in this?
- How did you experience the collaboration with the hospital in this process?
- What advice would you give to hospital staff to better prepare maternity nurses for families going home with a neonate on antibiotics?
- What advice would you give to other maternity nurses who may encounter this situation in the future?
