# Supplementary material for: Implementation strategy for an antibiotic stewardship bundle to promote optimal treatment choices in neonates with suspected early-onset sepsis (Protect-Neo): a study protocol for a multicentre, prospective interrupted time series and before-after study
Source: BMJ Open. 2025 Nov 4;15(11):e103368. doi: 10.1136/bmjopen-2025-103368 (PMC12588035; doi:10.1136/bmjopen-2025-103368)
Supplement: Supplementary file 10 [file bmjopen-2025-103368.reviewer_comments.pdf]

PEER REVIEW HISTORY

BMJ Open publishes all reviews undertaken for accepted manuscripts. Reviewers are asked to complete a checklist review form and are provided with free text boxes to elaborate on their assessment. These free text comments are reproduced below.

ARTICLE DETAILS

Title (Provisional)

Implementation strategy for an antibiotic stewardship bundle to promote optimal treatment choices in neonates with suspected early-onset sepsis (Protect-Neo): a study protocol for a multicentre, prospective interrupted time series and before-after study

Authors

van Veen, Liesanne E.J.; Tramper-Stranders, Gerdien A.; Achten, Niek B; Plötz, Frans B; van Rossum, Annemarie; Ista, Erwin

---

VERSION 1 - REVIEW

**Reviewer**	**1**
**Name**	**Ting, Joseph**
**Affiliation**	**University of Alberta**
**Date**	**22-Jun-2025**
**COI**	**None**

Congratulations on the development of this early-onset sepsis (EOS) study protocol. It represents a well-designed, prospective, multicentre implementation study with strong potential to inform clinical practice. I have a few minor suggestions for the team's consideration:

Recurrence of sepsis: It may be worthwhile to examine the recurrence of sepsis due to the same pathogen not only at the 28-day mark, but also at earlier intervals such as 7 and 14 days. These additional time points are valuable as balance indicators in quality improvement studies. Understanding whether early antibiotic exposure merely delays, rather than prevents, subsequent sepsis is also critical to interpreting outcomes meaningfully.

Microbiology and susceptibility patterns: It would be useful to document the microbiological profile and antimicrobial susceptibility of EOS pathogens, in order to assess whether the pattern of organism shifts following a reduction in antibiotic exposure.

**Reviewer**	**2**

**Name**	**Battersby, Cheryl**
**Affiliation**	**Imperial College London, Neonatal Medicine**
**Date**	**01-Sep-2025**
**COI**	**None**

I commend the authorship for publishing this protocol for ther implementation project in detail. Evaluating Quality improvement/ implementation projects in a robust manner on a scale involving 11 Dutch neonatal units is not a small undertaking.

My overall impresssion is that thevdescriptive elements of the bundle to b implemented, including all the stakeholders and steps have been very well thought through,however the quantiative analytical compontent needs more detail, and this is related to what the focus of the aims are.

Some queries:

1) The aims are written in a few different ways and I suggest this to be consistent and more succient in order to be able to be clear to the reader, what the aims of the project are.

I think the authors want to evaluate whether the care bundle reduces exposure to antibiotics and possibly length of stay? This means there is a comparison element that goes beyond description, but this is not well described in the statistical analytical methods section.

page 4 aim could be more succint and to the point - this sentence is far too long: "“Therefore, the aim of this study is to assess the impact of implementing a bundle of antibiotic stewardship interventions (including the EOS calculator, PCT-guided therapy and IV-to-oral switch therapy) in Dutch secondary hospitals and their surrounding first-line neonatal care networks, using a multicomponent implementation strategy, on both clinical and implementation outcomes, with a particular focus on evaluating the employed implementation strategies.”

2) What is the statistical plan to evaluate the impact of the implementation on the primary outcome given the pre-implementation phase, wash out period of implementation and post implementation? Can this be included please?

How will the statistical analysis deal with the before and after, secular trends to conclude there was a change? Would you be using a time series analysis/ joint point regression ?

How will you limit confounding in your analysis? What data / variables would you collect or use for this aspect?

3)For the qualitative methods: How will the study team ensure representative sample of parents/ staff agreeing to participate in the interviews/ focus groups? What information will you record of them?

4) some suggestions to clarify

It is not clear in the introduction or methods upfront which EOS calculator is being used.

It then is clearer later on it is the KP calculator; -Suggest to name which early onset sepsis calculator right you are using right upfront vs just referencing the website. The KP calculator is also due for an update and will I believe change soon, and apply only to infants 35 weeks and above. How this affect the study?

5) Minor suggestions :

- Limit the use of the word "Moreover" in the introduction

- What is a first-line neonatal care network and primary neonatal care ?

Would be useful to have some context to how the Dutch newborn care system works for international readers

US spelling specialized – does the journal need this changing to UK/British spelling?

VERSION 1 - AUTHOR RESPONSE

II. Reviewer: 1
Dr. Joseph Ting, University of Alberta
Comments to the Author:
Congratulations on the development of this early-onset sepsis (EOS) study protocol. It represents a well-designed, prospective, multicentre implementation study with strong potential to inform clinical practice. I have a few minor suggestions for the team’s consideration:

1. Recurrence of sepsis: It may be worthwhile to examine the recurrence of sepsis due to the same pathogen not only at the 28-day mark, but also at earlier intervals such as 7 and 14 days. These additional time points are valuable as balance indicators in quality improvement studies. Understanding whether early antibiotic exposure merely delays, rather than prevents, subsequent sepsis is also critical to interpreting outcomes meaningfully.

   Answer*: Thank you for your kind words and suggestions. We agree that sepsis delay or recurrence is valuable data to report, though the low prevalence of culture-positive sepsis may limit definitive conclusions. In our outcome measure, (re)infection within 28 days, we will include all recurrences occurring in this timeframe. For any (re)infection, we will collect detailed case data and report these findings accordingly. This approach will allow us to address outcomes at several time points within the first month.*

   When reevaluating our outcomes in light of your comment, we realized that our initial definition was too narrow. Initially, we defined (re)infection and readmission in relation to therapy completion. However, with our bundle of intervention, many infants will be managed by observation rather than immediate treatment. It is therefore relevant to also capture those who were initially observed but later return with an infection. For this reason, we have redefined the outcome from the time of birth, ensuring that all reinfections are included—whether after treatment or after an observation period.

2. Microbiology and susceptibility patterns: It would be useful to document the microbiological profile and antimicrobial susceptibility of EOS pathogens, to assess whether the pattern of organism shifts following a reduction in antibiotic exposure.

Answer: *Thank you for this suggestion. We recognize the added value of reporting pathogen profiles and susceptibility patterns and will include these data in the supplementary materials of the final manuscript. We have added this to the description of our secondary outcomes. However, the low prevalence of culture-positive EOS may limit assessing shifts. Moreover, as our protocol focuses on early-onset sepsis, typically caused by vertically transmitted pathogens and treated within 72 hours of birth, we do not expect substantial shifts in organism distribution or susceptibility. Such shifts will more likely manifest in later infections, such as late-onset sepsis, which are outside the scope of our study.*

<u>Modified version line 318-324</u>
We will determine the proportion of neonates starting on antibiotics per 100 live births, proportion of neonates receiving antibiotics per predefined category of duration (36 hours, 36-48 hours, 48-72, >5 days and >7days), duration of therapy, proportion of neonates receiving IV-to-oral switch therapy, duration of hospital stay, cumulative number of laboratory tests, cumulative readmission rate within 28 days after birth, cumulative bacterial (re)infection rate within 28 days after birth, incidence of culture-proven EOS per 1000 live births, pathogen determination and antibiotic susceptibility, and infection related mortality within 28 days after birth.

Reviewer: 2
Dr. Cheryl Battersby, Imperial College London
Comments to the Author:
I commend the authorship for publishing this protocol for theirther implementation project in detail. Evaluating Quality improvement/ implementation projects in a robust manner on a scale involving 11 Dutch neonatal units is not a small undertaking.

Answer: *Thank you for your kind comment and recognition for the work put into this study.*

My overall impresssion is that the descriptive elements of the bundle to b implemented, including all the stakeholders and steps have been very well thought through, however the quantiative analytical compontent needs more detail, and this is related to what the focus of the aims are.
Some queries:

1. The aims are written in a few different ways and I suggest this to be consistent and more succient in order to be able to be clear to the reader, what the aims of the project are.
   I think the authors want to evaluate whether the care bundle reduces exposure to antibiotics and possibly length of stay? This means there is a comparison element that goes beyond description, but this is not well described in the statistical analytical methods section.

   page 4 aim could be more succint and to the point - this sentence is far too long: "“Therefore, the aim of this study is to assess the impact of implementing a bundle of antibiotic stewardship interventions (including the EOS calculator, PCT-guided therapy and IV-to-oral switch therapy) in Dutch secondary hospitals and their surrounding first-line neonatal care networks, using a multicomponent implementation strategy, on both clinical and implementation outcomes, with a particular focus on evaluating the employed implementation strategies.”

Answer: *Thank you for this suggestion. We agree the aims should be aligned and the mentioned sentence is too long. We have rewritten the aim in the abstract and introduction section and aimed to describe our study’s goal clearer. We think this now also aligns with our primary outcome measure: antibiotic exposure measured in DOTs per 1000 live births.*

<u>Modified version line 22-25</u>
The primary aim of this study is to assess the impact of implementing a bundle of antibiotic stewardship interventions, including the early-onset sepsis calculator, procalcitonin-guided

therapy and IV-to-oral switch therapy on antibiotic exposure in Dutch secondary hospitals. Secondary aims are to examine additional clinical outcomes and implementation outcomes.

Modified version line 96-100
Therefore, the primary aim of this study is to assess the impact of the strategy to implementing an antibiotic stewardship bundle (the EOS calculator, PCT-guided therapy and IV-to-oral switch therapy) on antibiotic exposure for EOS in Dutch secondary hospitals. Secondary aims are to assess additional clinical outcomes and implementation outcomes, including the acceptability, feasibility and fidelity of the interventions, as well as the appropriateness of the implementation strategies.

2. What is the statistical plan to evaluate the impact of the implementation on the primary outcome given the pre-implementation phase, wash out period of implementation and post implementation? Can this be included please?
   How will the statistical analysis deal with the before and after, secular trends to conclude there was a change? Would you be using a time series analysis/ joint point regression?
   How will you limit confounding in your analysis? What data / variables would you collect or use for this aspect?

   Answer: *Thank you for this important comment. We have carefully reevaluated the statical plan and agree that a time series analysis is more appropriate. For our primary outcome, days on therapy per 1000 livebirths, we will perform an interrupted time series regression (ITS), comparing the pre- and post-implementation periods while taking the existing trends into account. We will perform a multilevel ITS to account for differences in DOT at baseline and differences in trends overtime. We will use 4 timepoints (units of 3 months) in both the pre- and post-implementation period, resulting in a total of 8 timepoints. We know that a higher number is preferred, yet as EOS is relatively rare, the number of cases and thus treatment are prone to fluctuations. However, if the data allows for it, we will use more timepoints. We added a description of our planned analysis to the 'statistical analysis' part of the method section. We also added information in our manuscripts' title, abstract and in the description of our study design in the method section, to make this consequent throughout the entire manuscript.*

   Modified version line 408-426
   An interrupted time series analysis (ITS) will be conducted, to compare trend of overall DOT (all types of antibiotic agents) per 1000 live births pre- and post-intervention. The ITS will have at least four data points in the pre-implementation period and four datapoints in the post-implementation period, based on quarterly intervals. If the number of cases allows dividing the data into more datapoints, this may be considered. To account for potential clustering of observations within hospitals and potential differences between hospitals, we will apply a mixed-effects segmented regression analysis for ITS ('joinpoint regression') with DOT as dependent variable, and random intercept for each hospital. Also, we will include **random slopes for the post-intervention trend,** allowing hospitals to differ not only in baseline DOT levels but also in hospitals respond differently to the implementation. Fixed effects include intervention phase (pre/post) and segmented time after the intervention to estimate both immediate level changes and changes is trend. Confounders, including the number of positive blood cultures, mean gestational age and mean birth weight will be included as time-varying covariates. Depending on data availability, we may also examine trends during the intermediate **implementation phase** to explore changes during this transition period as secondary analysis. Autocorrelation of residuals will be checked by using the Durbin-Watson test, and if present, corrected. Results will be reported as adjusted changes in level and trend, with 95% confidence intervals, reflecting both pooled effects and between-hospital variability. Additionally, a before-after comparison will be performed using a t-test or Mann-Whitney U test, for both overall DOT and DOT per individual antibiotic agent.

3. For the qualitative methods: How will the study team ensure representative sample of parents/ staff agreeing to participate in the interviews/ focus groups? What information will you record of them?

   Answer: *Thank you for highlighting this point. We added information on the sampling method for the focus groups and for the data recorded of participants in all the types of qualitative research. The sampling of parents and maternity nurses for interviews however is a challenge, because of the relative rarity of neonates that receive oral therapy (which is the focus of these interviews). Our priority lies with recruiting enough participants to reach data saturation. To enhance representativeness, we will interview parents from all 11 sites, thus from different Dutch regions. We did however not get consent to record their ethnicity or socioeconomic status. For the maternity nurses we will use 'snowball' sampling, to identify those maternity nurses that were exposed to oral switch therapy, as most Dutch maternity nurses are not yet involved in this care. With these approaches we aim to balance methodological rigor with practical constraints, supporting the collection relevant qualitative data.*

   Added text line 360-365
   To ensure a sample that is as representative as possible, all focus groups will be conducted during regularly scheduled ward meetings during working hours, typically attended by all physicians on duty. These meetings naturally include a diverse group of individuals with varying roles and expertise within the department. Those expected to be on duty on the day of the focus group will be invited to participate by the local champions. Participants' gender, professional role and years of working experience in their role will be recorded.

   Added text line 376-375
   Data on parents' gender, age and number of children will be recorded.

   Added text line 382-383
   Data on maternity nurses' gender and years of working experience will be recorded.

4. Some suggestions to clarify
   It is not clear in the introduction or methods upfront which EOS calculator is being used.
   It then is clearer later on it is the KP calculator; -Suggest to name which early onset sepsis calculator right you are using right upfront vs just referencing the website. The KP calculator is also due for an update and will I believe change soon, and apply only to infants 35 weeks and above. How this affect the study?

   Answer: *We agree that it should be clear to all readers which calculator is being used. To address this point, we clarified in the introduction and methods which calculator is applied and provided a reference to the original KP research article. The point regarding the updated KP calculator is indeed important, as the update currently took place and changed the website. As this 2025 version, which is based on a new cohort and universal GBS screening, is not validated for the Dutch population, we will use the 2017 version, and it will thus not influence our study.*

   Modified version line 67-69
   Firstly, utilization of the EOS calculator, developed by Kaiser Permanente, has shown to reduce up to 44% of antibiotic prescriptions in neonates at increased risk of EOS compared to the conventional risk categorization approach [10,15,16].

   Modified version line 134-135
   The EOS calculator, which was developed by researchers of Kaiser Permanente, is applicable to neonates born at 34 weeks of gestation or later and can be used within the first 24 hours after birth [10].

5. Minor suggestions :
   - Limit the use of the word "Moreover"   in the introduction

   Answer: *We indeed noticed the word 'moreover' being used twice shortly after each other. We deleted the second 'moreover' in line 96.*

What is a first-line neonatal care network and primary neonatal care ?
Would be useful to have some context to how the Dutch newborn care system works for international readers

Answer: *It is indeed important to clarify the Dutch newborn system for our readers. Firstly, we changed the wording to primary care in the entire manuscript, as this is the same as 'first-line', but only described differently. Secondly, we added a concise explanation of the primary neonatal care in the 'study setting' part of the method section.*

<u>Added part line 117-123</u>
In the Netherlands, neonates at risk for early-onset sepsis (EOS) are initially managed either in obstetric wards (Level I) or neonatal wards (Levels II–IV), depending on their age and clinical condition. Most late-preterm and term neonates receive care in secondary hospitals (Level I-II facilities). Following discharge, continuity of care is provided by midwives, maternity nurses, and general practitioners. Midwives hold medical responsibility for the mother and clinically well-appearing neonates during the first 10 days postpartum, with support from maternity nurses in the home setting. In cases where neonates continue oral antibiotic therapy at home, medical responsibility remains with the paediatrician, while supported by primary care.

6. US spelling specialized – does the journal need this changing to UK/British spelling?

   Answer*: In line with the journal requirements, we changed the text to UK spelling.*
